# G protein-biased LPAR1 agonism of prototypic antidepressants: Implication in the identification of novel therapeutic target for depression

**DOI:** 10.1038/s41386-023-01727-9

**Published:** 2023-09-06

**Authors:** Naoto Kajitani, Mami Okada-Tsuchioka, Asuka Inoue, Kanako Miyano, Takeshi Masuda, Shuken Boku, Kazuya Iwamoto, Sumio Ohtsuki, Yasuhito Uezono, Junken Aoki, Minoru Takebayashi

**Affiliations:** 1 Department of Neuropsychiatry, Faculty of Life Sciences, Kumamoto University, Kumamoto, 860-8556 Japan; 2 Center for Metabolic Regulation of Healthy Aging, Faculty of Life Sciences, Kumamoto University, Kumamoto, 860-8556 Japan; 3 Division of Psychiatry and Neuroscience, Institute for Clinical Research, National Hospital Organization Kure Medical Center and Chugoku Cancer Center, Kure, 737-0023 Japan; 4 Laboratory of Molecular and Cellular Biochemistry, Graduate School of Pharmaceutical Sciences, Tohoku University, Sendai, 980-8578 Japan; 5 Department of Pain Control Research, The Jikei University School of Medicine, Tokyo, 105-8461 Japan; 6 Division of Cancer Pathophysiology, National Cancer Center Research Institute, Tokyo, 104-0045 Japan; 7 Department of Pharmaceutical Microbiology, Faculty of Life Sciences, Kumamoto University, Kumamoto, 862-0973 Japan; 8 Department of Molecular Brain Science, Graduate School of Medical Sciences, Kumamoto University, Kumamoto, 860-8556 Japan; 9 Department of Health Chemistry, Graduate School of Pharmaceutical Sciences, The University of Tokyo, Tokyo, 113-0033 Japan

**Keywords:** Pharmacology, Depression, Neuroscience, Drug development

## Abstract

Prototypic antidepressants, such as tricyclic/tetracyclic antidepressants (TCAs), have multiple pharmacological properties and have been considered to be more effective than newer antidepressants, such as selective serotonin reuptake inhibitors, in treating severe depression. However, the clinical contribution of non-monoaminergic effects of TCAs remains elusive. In this study, we discovered that amitriptyline, a typical TCA, directly binds to the lysophosphatidic acid receptor 1 (LPAR1), a G protein-coupled receptor, and activates downstream G protein signaling, while exerting a little effect on β-arrestin recruitment. This suggests that amitriptyline acts as a G protein-biased agonist of LPAR1. This biased agonism was specific to TCAs and was not observed with other antidepressants. LPAR1 was found to be involved in the behavioral effects of amitriptyline. Notably, long-term infusion of mouse hippocampus with the potent G protein-biased LPAR agonist OMPT, but not the non-biased agonist LPA, induced antidepressant-like behavior, indicating that G protein-biased agonism might be necessary for the antidepressant-like effects. Furthermore, RNA-seq analysis revealed that LPA and OMPT have opposite patterns of gene expression changes in the hippocampus. Pathway analysis indicated that long-term treatment with OMPT activated LPAR1 downstream signaling (Rho and MAPK), whereas LPA suppressed LPAR1 signaling. Our findings provide insights into the mechanisms underlying the non-monoaminergic antidepressant effects of TCAs and identify the G protein-biased agonism of LPAR1 as a promising target for the development of novel antidepressants.

## Introduction

Antidepressants have been developed to increase monoamine selectivity, as evidenced by serotonin and noradrenaline reuptake inhibitors (SNRIs) and selective serotonin reuptake inhibitors (SSRIs). While SNRIs and SSRIs have reduced side effects and increased tolerability compared to prototypic antidepressants, such as tricyclic/tetracyclic antidepressants (TCAs), their therapeutic effects may be comparable or even lower than those of TCAs [1, 2]. As approximately 30% of depression is treatment-resistant, antidepressants with pharmacological actions other than increasing monoamine selectivity need to be developed. Tricyclic antidepressants have been reported to be more effective than SSRIs in treating severe depression [2]; however, the mechanisms underlying their therapeutic effects remain unclear. These reports have suggested that, in addition to modulating monoaminergic neurotransmission, TCAs may act on other therapeutic targets. In this study, we hypothesized that one of the potential targets of TCAs is the lysophosphatidic acid receptor 1 (LPAR1), a G protein-coupled receptor (GPCR). LPAR1 is one of the six receptors (LPAR1–6) that are activated by lysophosphatidic acid (LPA), a bioactive phospholipid. The receptor is activated by TCAs [3–5] and contributes to emotional behaviors [6, 7]. LPAR1-deficient mice exhibited abnormalities in hippocampal functions and showed a phenotype with depressive and anxious features [7, 8]. However, such a depression-like phenotype was also observed when LPA was administered into rodent brains [9, 10], which seems to contradict our hypothesis.

LPA binding to LPAR1 not only stimulates G protein signaling but also promotes receptor phosphorylation by G protein-coupled receptor kinases and subsequent binding of β-arrestins, which in turn mediates endocytosis and receptor desensitization [11, 12]. Although GPCRs typically activate both G protein signaling and β-arrestin-mediated endocytosis, some GPCR ligands preferentially activate either of the signaling pathways, a phenomenon referred to as biased agonism [13]. For instance, fingolimod, a β-arrestin-biased agonist of sphingosine 1-phosphate receptor, is a functional antagonist, as it selectively induces desensitization via β-arrestin-mediated endocytosis [14]. LPA may cause functional antagonism of LPAR1 via endocytosis [15], potentially blocking LPAR1 signaling, as observed in LPAR1-deficient mice. Therefore, we sought to determine whether TCAs activate downstream signals of LPAR1 similar to LPA, or whether they activate selective signals distinct from LPA. Additionally, we examined whether LPAR1 may be involved in mediating the behavioral effects of TCAs.

## Materials and Methods

### Affinity purification of LPAR1 with TCA-immobilized magnetic beads (TCA-beads)

The affinity purification of LPAR1 from the LPAR1-overexpressing membrane lysates was performed using TCA-beads according to standard protocols (see supplementary methods).

### Immunoblotting

Immunoblotting was performed using standard protocols (see supplementary methods).

### Transforming growth factor α (TGFα) shedding assay

The TGFα shedding assay, which measures the activation of specific GPCR-dependent G protein signaling, was performed as described previously [16], with minor modifications (see supplementary methods).

For LPA treatment, we used 18:1 LPA (1-oleoyl-2-hydroxy-sn-glycero-3-phosphate (sodium salt); CAS No. 325465-93-8) throughout all experiments in this study.

### NanoBiT-based β-arrestin1 recruitment assay

The NanoBiT protein-protein interaction assay-based β-arrestin1 recruitment assay was performed as described previously [17], with minor modifications (see supplementary methods).

### Animals

Male C57BL/6J mice and LPAR1-heterozygous mice (B6N(Cg)-Lpar1<tm1b(EUCOMM)Wtsi>/J, Stock No.27468) were used in this study; the mice were 7–8 weeks old at the beginning of the experiments. Mice were obtained from The Jackson Laboratory Japan (Yokohama, Japan) and maintained with a 12 h light/dark cycle with food and water ad libitum at a controlled temperature (23–25 °C). All experimental procedures were performed in accordance with the Guideline for Animal Experiments in the National Hospital Organization Kure Medical Center and Chugoku Cancer Center (NHOKMCCCC). Protocols were approved by the Animal Research Ethics Committee, NHOKMCCCC (Approval No. 2019-01 and 2020-03).

### Behavioral procedures

The forced swim test (FST), open field test (OFT) and sucrose preference test (SPT) were performed using standard protocols (see supplementary methods).

### Drug treatment timeline

For subchronic amitriptyline treatment, amitriptyline was dissolved in tap water at a concentration of 160 mg/L. The average intake dose of amitriptyline per mouse during the experiment was 12.99 ± 0.19 mg/kg/day based on the average amount of water consumed and the average weight of the mice used in this study. Before its administration, an FST was performed to measure the basal immobility time. Mice were then grouped to ensure that there was no bias in basal immobility time. Subsequently, an FST was performed at 1 and 2 weeks after administration.

For chronic corticosterone (CORT) treatment, mice were treated with CORT (35 mg/L, Tokyo Chemical Industry) or 0.45% hydroxypropyl-β-cyclodextrin (Fujifilm Wako, Osaka, Japan), added to their drinking water, for 7 weeks. The average intake dose of CORT per mouse during the experiment was 7.29 ± 0.68 mg/kg/day. During the last 3 weeks of CORT treatment, mice were injected daily with amitriptyline (10 mg/kg/day, i.p.) and/or Ki16425 (10 mg/kg/day, i.p.), and an SPT was performed subsequently.

For continuous intrahippocampal injection, mice were anesthetized with isoflurane and surgically implanted with two subcutaneous osmotic minipumps (Alzet model 1004; Durect Corporation, Cupertino, CA, USA) and bilateral guide cannulae (Plastics One, Roanoke, VA, USA) targeting hippocampi. The minipumps were filled with LPA (15 nM in PBS), OMPT (15 nM in 40% DMSO and 60% PBS), PBS (vehicle for LPA), or 40% DMSO-PBS (vehicle for OMPT), and activated the evening before surgery by incubating them at 37 °C in saline to initiate a continuous delivery at 0.11 μL/h over 2 weeks. Bilateral cannulae were delivered into the hippocampus at –2.2 mm posterior to the bregma, ± 1.5 mm lateral to the midline, and –2.0 mm ventral to the surface of the skull. The antibiotic penicillin G (500 units/mouse, i.m.) and the analgesic carprofen (5 mg/kg, i.p.) were administered after surgery. Before surgery, an FST was performed to measure the basal immobility time. Mice were grouped to ensure that there was no bias in basal immobility time. Subsequently, an FST was performed at 1 and 2 weeks after the surgery, followed by an OFT.

### RNA-seq and data analysis

Total RNA was extracted from frozen whole hippocampi using the AllPrep DNA/RNA mini kit (Qiagen, Hilden, Germany) according to the manufacturer’s instructions. Library preparation and RNA-seq were outsourced to Macrogen Japan (Kyoto, Japan; see supplementary methods). For data analysis, raw reads were trimmed using Trimmomatic (v0.39) and then aligned to the mm10 reference genome using STAR (v2.7.9a). Gene expression was quantitated using RSEM (v1.3.3) and TCC-GUI [18] was used to determine the differentially expressed genes. Threshold-free genome-wide transcriptomic overlap analysis was conducted using rank-rank hypergeometric overlap (RRHO2, v1.0). Canonical pathways were generated using Ingenuity Pathway Analysis (IPA, Release Date: 2021-10-22, Qiagen; see supplementary methods).

### Statistical analysis

All data are presented as mean ± SEM. Statistical significance was determined using various methods depending on the experimental design. For parametric data sets, we employed one-way ANOVA. For post hoc analysis of one-way ANOVA, we employed Dunnett’s multiple comparisons test when comparing multiple groups against a control group. Tukey’s multiple comparisons test was used for situations where all groups were compared to each other. Sidak’s multiple comparisons test was employed when comparing only selected groups. For comparisons between two groups, the unpaired t-test was used. Two-way ANOVA was employed for experiments involving two independent variables. In cases where data did not meet parametric assumptions, the Kruskal-Wallis test was used, followed by post hoc Dunn’s multiple comparisons test. For experiments involving repeated measurements over time, we used mixed-effects models with post hoc Sidak’s multiple comparisons test. All statistical analyses were performed using GraphPad Prism 9 software. In the figures, significant effects are denoted by asterisks that indicate *P < 0.05, **P < 0.01, and ***P < 0.001.

## Results

### TCAs directly bound to LPAR1 and competitively acted with LPA

**Fig. 1:**
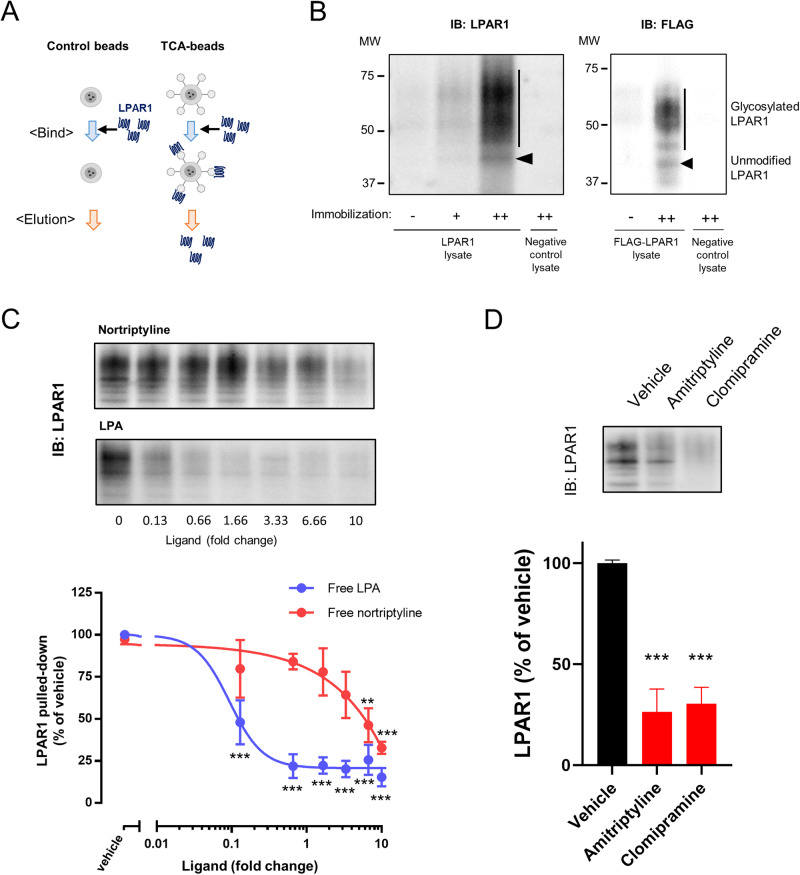
Tricyclic antidepressants directly bind to LPAR1. **A** Scheme of affinity purification experiment of LPAR1 with TCA-beads. **B** Immunoblots from affinity-purified lysates using TCA-beads or control beads. The amount of nortriptyline immobilized on the beads was 19.3 nmol/mg (+) or 27.3 nmol/mg (++). Two cell lysates were used for affinity purification, namely lysates from human LPAR1-overexpressed RH7777 cells (left) and those from FLAG-tagged human LPAR1-transfected HeLa cells (right). Each parent cell lysate was used as a negative control lysate. **C** Competitive inhibition of LPAR1 binding to TCA-beads. LPAR1-overexpressed RH7777 cell lysates were preincubated with the indicated concentrations of nortriptyline or LPA (shown as a ratio to the amount of nortriptyline immobilized on the TCA-beads) and then eluted with TCA-beads. Representative immunoblots are shown above the graph. **D** Amitriptyline and clomipramine (6.67-fold concentration relative to the amount of nortriptyline immobilized on the TCA-beads) inhibit LPAR1 binding to TCA-beads. LPAR1-overexpressed RH7777 cell lysates were used. A representative immunoblot is shown above the graph. *N* = 3–4. Data are presented as the means ± SEM. Statistical significance was calculated using one-way ANOVA with Dunnett’s multiple comparisons test (**P < 0.01, ***P < 0.001 vs vehicle).

To investigate whether TCA binds to LPAR1 directly, affinity purification experiments [19] were performed with TCA-beads (schematic representation: Fig. 1A). We attempted to create TCA-beads with amitriptyline, a typical TCA. However, amitriptyline did not have the functional group required for the immobilization with the N-hydroxysuccimide (NHS)-magnetic beads (NHS-beads). Therefore, nortriptyline, which has the same structure as amitriptyline except for the secondary amine, was immobilized (Fig. S1). In this study, nortriptyline-immobilized beads are defined as “TCA-beads.” Unmodified forms of LPAR1 (41 kDa) as well as glycosylated forms of LPAR1 (50–75 kDa) were detected in the LPAR1-overexpressing cell lysates (Fig. 1B). TCA-beads eluted LPAR1 depending on the amount of immobilization. However, TCA-beads did not elute LPAR1 from negative control lysate (Fig. 1B). Next, we investigated whether the binding between TCA-beads and LPAR1 was competitively inhibited by using an excessive amount of free ligand. The maximum concentration of competitive ligand was set to 10 times the amount of nortriptyline on the beads (0.75 mM). Free nortriptyline, the same ligand immobilized on the beads, inhibited the binding between LPAR1 and the TCA-beads in a concentration-dependent manner (Fig. 1C). Moreover, free LPA, an endogenous agonist of LPAR1, also inhibited the binding at lower concentrations than free nortriptyline (Fig. 1C). It is likely that this assay detects the higher affinity of LPA for LPAR1 than nortriptyline. As the results of such a strategy have been shown previously [20], a competitive assay with free test ligands would likely reveal the direct binding of the ligand to LPAR1. Finally, other free TCAs, such as amitriptyline and clomipramine, also significantly inhibited the binding process (Fig. 1D), suggesting that TCAs directly bind to LPAR1.

A previous study using an electrical impedance (ΔZ)-based biosensor reported that amitriptyline activated G protein (increase in ΔZ) via endogenous LPAR1 in label-free C6 cells [3]. LPA significantly increased ΔZ in a dose-dependent manner, which was saturated above 3 μM (Fig. S2A). An additive effect by 25 μM of amitriptyline was observed in the low dose range (<1 μM) of LPA, but not above the saturated dose of LPA (≧3 μM) (Fig. S2A). DAMGO, a ligand for μ-opioid receptors (MOR), also increased ΔZ in a dose-dependent manner in stable MOR-expressing HEK293 cells (Fig. S2B). Moreover, 25 μM of amitriptyline showed a significant additive effect even at a saturated dose of DAMGO (≧1 μM) (Fig. S2B), suggesting that amitriptyline and DAMGO are mediated by distinct receptors. These findings suggest that amitriptyline directly interacts with endogenous LPAR1 in living cells.

### TCAs, but not other types of antidepressants, acted as a G protein-biased LPAR1 agonist

**Fig. 2:**
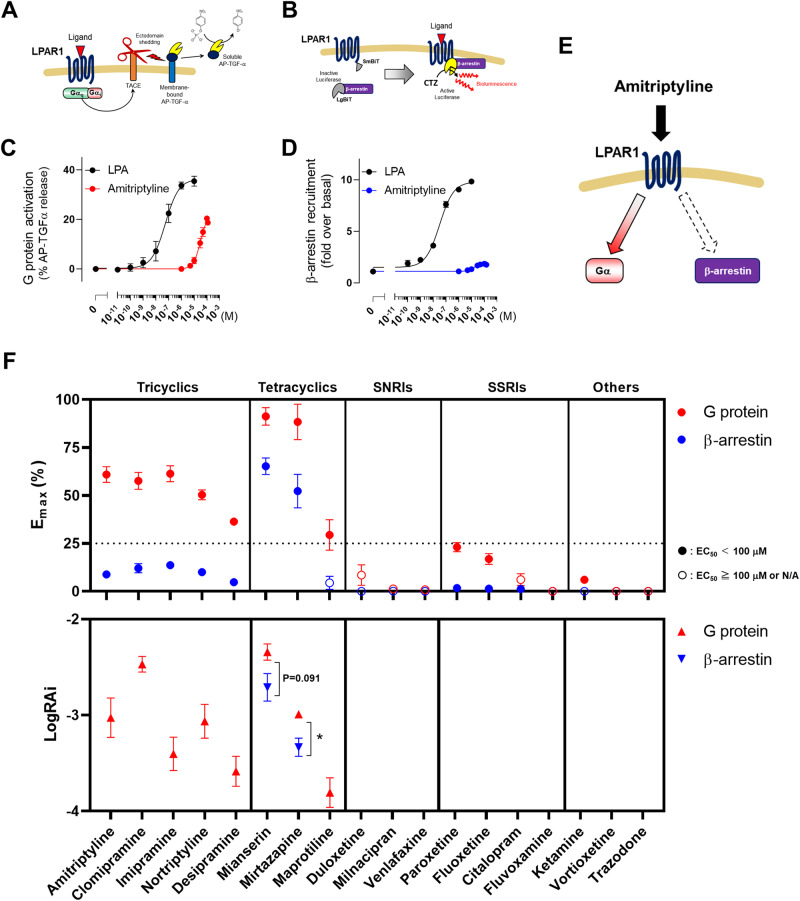
Tricyclic and tetracyclic antidepressants, but not other antidepressants, are G protein-biased LPAR1 agonists. **A** Schematic representation of TGFα shedding assay and **B** β-arrestin recruitment assay. Dose-response curves of LPA and amitriptyline for the LPAR1-specific **C** G protein activation and **D** β-arrestin recruitment. *N* = 3–4. Data are presented as means ± SEM. **E** Possible LPAR1 downstream pathways activated by amitriptyline. **F** E_max_ values (top; E_max_ of LPA = 100%) for each antidepressant calculated from dose-response curves and LogRAi values (bottom) for each antidepressant that showed agonist activity (E_max_ > 25% and EC_50_ < 100 μM). *N* = 3–4. Data are presented as the means ± SEM. *P < 0.05 (Unpaired *t*-test).

To investigate whether antidepressants have a signaling bias downstream of LPAR1, we measured G protein signaling and β-arrestin response via TGFα shedding assay [16] (Fig. 2A) and NanoBiT-based β-arrestin recruitment assay [17] (Fig. 2B), respectively. LPA was used as a reference. Amitriptyline treatment showed LPAR1-specific G protein signaling activity (E_max_ = 60.9 ± 4.1%), but less β-arrestin recruitment to LPAR1 (E_max_ = 8.8 ± 1.9%) (Fig. 2C, D), indicating that amitriptyline is biased towards activating G protein signaling (Fig. 2E). Further, we examined whether different types of antidepressants could activate LPAR1 downstream pathways (Fig. S3). Considering that antidepressants can accumulate at concentrations of tens of micromolars in the brain [21–23], antidepressants with E_max_ > 25% and EC_50_ < 100 μM were defined as agonists in this study. To quantitatively evaluate the potency of individual signaling activities of each agonist, we used relative intrinsic activity (RAi) values [24, 25]. All tested TCAs showed agonist activity for G protein signaling, whereas the other tested antidepressant drugs, including SNRIs, SSRIs, ketamine, vortioxetine, or trazodone, did not (Fig. 2F). Mianserin and mirtazapine showed agonist activity for β-arrestin signaling but their G protein signaling activities were significantly higher or tended to be higher than their β-arrestin signaling activities (Fig. 2F). Results suggested that the G protein-biased agonism at LPAR1 is unique to TCAs.

### LPAR1 mediated the behavioral effects of amitriptyline

**Fig. 3:**
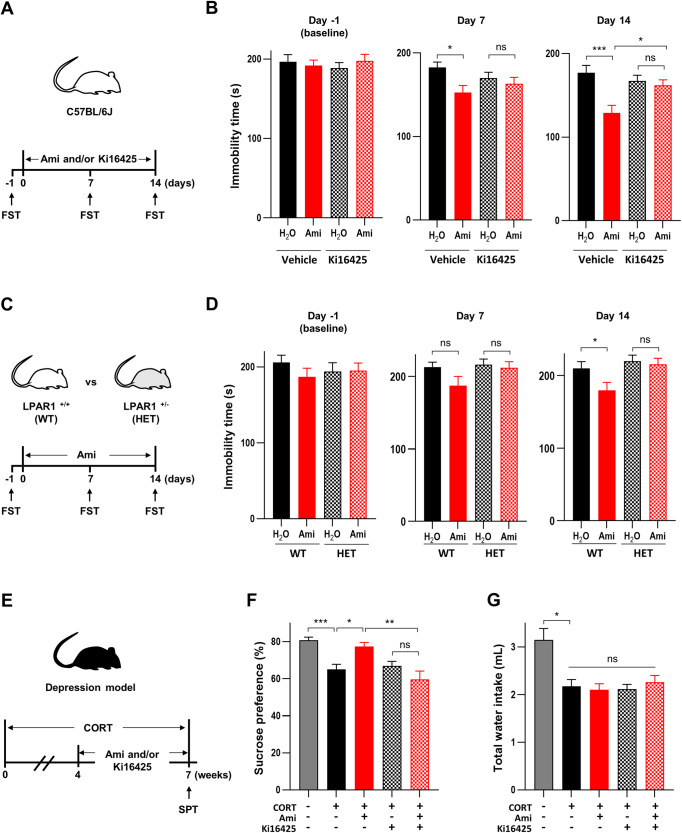
LPAR1 mediates the behavioral effects of amitriptyline (Ami). **A** Timeline and group design of the experiment is shown in panel **B**. **B** Immobility time in the forced swim test (FST). Mice were treated with Ami (160 mg/L), added to their drinking water, and were injected daily with Ki16425 (10 mg/kg/day, i.p.) or vehicle for 14 days. Repeated FST was performed on Day –1, 7, and 14. *N* = 36. Data are presented as means ± SEM. Statistical significance was calculated using one-way ANOVA with Sidak’s multiple comparisons test (*P < 0.05, ***P < 0.001, ns: not significant). **C** Timeline and group design of the experiment shown in panel **D**. **D** Immobility time in the FST. Wild type (WT) and LPAR1 heterozygous (HET) mice were treated with Ami (160 mg/L) in drinking water for 14 days. Repeated FST was performed on days –1, 7, and 14. *N* = 22 (vehicle in WT), 19 (Ami in WT), 17 (vehicle in HET), and 16 (Ami in HET). Statistical significance was calculated using one-way ANOVA with Sidak’s multiple comparisons test (*P < 0.05, ns: not significant). **E** Timeline and group design of the corticosterone (CORT)-treated mice. **F** Percentage of sucrose preference and **G** total water intake. Mice were treated with CORT (35 mg/L) or vehicle (Veh), added to their drinking water, for 7 weeks. During the last 3 weeks of CORT treatment, mice were injected daily with amitriptyline (10 mg/kg/day, i.p.) and/or Ki16425 (10 mg/kg/day, i.p.). *N* = 15 (vehicle and CORT+Ki16425) and 17 (CORT, CORT+Ami, and CORT+Ami+Ki16425). Statistical significance was calculated using the Kruskal–Wallis test with Dunn’s multiple comparisons test (*P < 0.05, ***P < 0.001, ns: not significant).

Next, we determined whether LPAR1 is required for the behavioral effects of amitriptyline using the FST, a commonly used method for evaluating the effects of antidepressants [26]. Acute treatment with amitriptyline decreased immobility in the FST, indicating an antidepressant effect, which was blocked by NAN-190, a 5HT1A antagonist, but not blocked by Ki16425, an LPAR1–3 antagonist (Fig. S4A). We then conducted repeated FSTs to assess the effects of antidepressants over time in the same animals [27, 28]. One- and two-week treatment with amitriptyline significantly reduced immobility time, which could be blocked by Ki16425 (Fig. 3A, B). These results suggest that LPARs may be involved in the long-term effects of amitriptyline.

We also conducted the FST for fluoxetine, a typical SSRI. Similar to amitriptyline, acute administration of fluoxetine resulted in a decrease in immobility, which could be attributed to 5HT1A involvement rather than LPARs (Fig. S4B). In contrast, two weeks of fluoxetine treatment led to an increase in immobility, regardless of whether it was combined with Ki16425 (Fig. S5).

To confirm the pharmacological results, we used genetically modified LPAR1 heterozygous mice in this study. C57BL/6 J background LPAR1-deficient mice were deemed unsuitable for behavioral experiments, as they exhibited neonatal mortality and weight loss (Fig. S6), which is consistent with a previous report [29]. The expression level of LPAR1 in the brain of these mice was found to be reduced by half compared to wild-type mice [30]. Like the results from Ki16425 treatment, LPAR1 heterozygous mice showed no effect of subchronic amitriptyline administration in the FST (Fig. 3C, D). To assess this with another behavioral test, we employed the SPT using a CORT-induced mouse model of depression [31]. Three weeks of amitriptyline treatment normalized the CORT-induced decrease in sucrose preference, an indicator of anhedonia, but had no effect in mice co-treated with Ki16425 (Fig. 3E, F). Administration of CORT decreased total water intake during the SPT, but neither amitriptyline nor Ki16425 affected the intake (Fig. 3G), suggesting that the changes in preference induced by amitriptyline and Ki16425 were not due to changes in intake. These results indicate that LPAR1 is involved in the behavioral changes induced by subchronic amitriptyline treatment.

### Quantification of hippocampal monoamines in heterozygous LPAR1 knockout mice after subchronic amitriptyline treatment

To investigate the effect of LPAR1 signaling on hippocampal monoamines, we analyzed hippocampal monoamine content. Heterozygous LPAR1 knockout mice exhibited no significant changes in hippocampal monoamine levels compared to wild-type mice, regardless of a 2-week amitriptyline treatment (Fig. S7). These findings suggest that the consistent decrease in LPAR1 signaling may not exert substantial influence on hippocampal monoamine levels, regardless of the presence or absence of antidepressant treatment.

### Characterization of LPAR1 downstream signaling pathways using various LPAR1 agonists

**Fig. 4:**
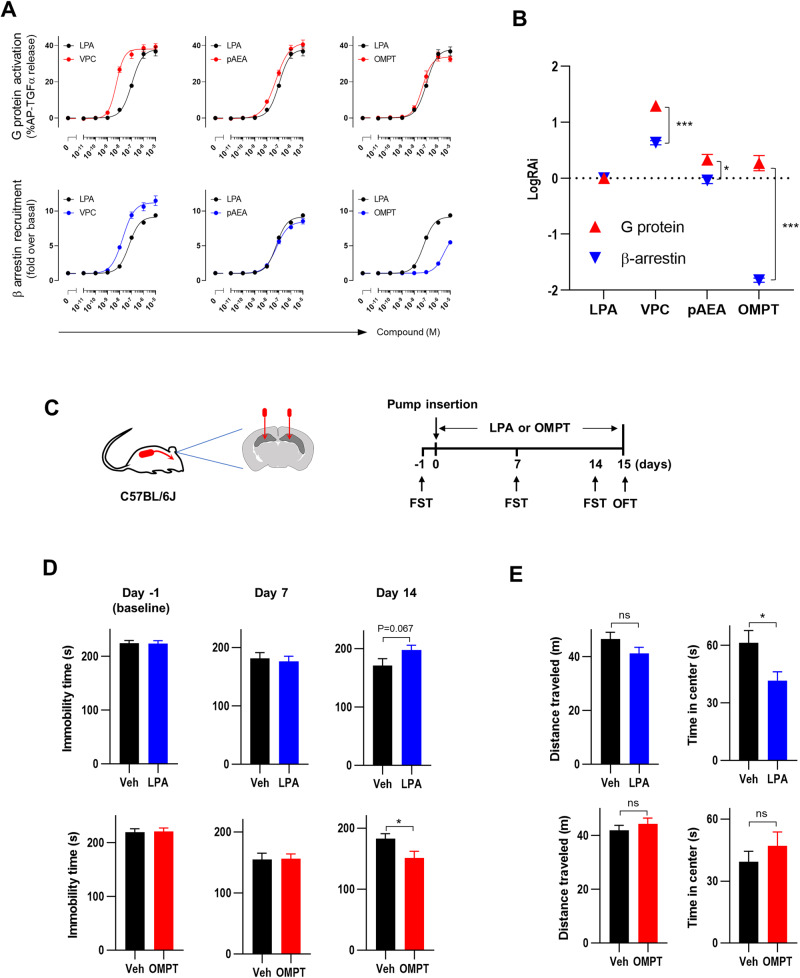
G protein-biased LPAR1 agonist induces antidepressant-like effects. **A** Dose-response curves and **B** logRAi values of LPAR1 agonists (LPA, VPC, pAEA, and OMPT) for the LPAR1-specific G protein activation and β-arrestin recruitment. *N* = 3. Data are presented as means ± SEM. *P < 0.05, ***P < 0.001 (Unpaired *t*-test). **C** Timeline of the experiments using mice treated with LPA or OMPT intrahippocampally. **D** Immobility time in the forced swim test (FST) and **E** time in the center and distance traveled in the open field test (OFT). OMPT or LPA was continuously infused into the hippocampi for 2 weeks using osmotic pumps (concentration in pump: 15 nM, delivery rate: 0.11 μL/h). Repeated FST was performed on days –1, 7, and 14, and the OFT was performed on Day 15. *N* = 24. *P < 0.05, ns: not significant (Unpaired *t*-test).

We examined the effects of commercially available LPAR1 agonists, including VPC31143, phospho-anandamide (pAEA), and OMPT, on the downstream signaling of LPAR1. They showed agonistic activities in both TGFα shedding and β-arrestin recruitment assays (Fig. 4A). Among the three agonists, OMPT exhibited a greater decrease in β-arrestin signaling than in G protein signaling (Fig. 4B).

LPA treatment decreased the cell surface expression of LPAR1 on HEK293 cells, whereas it did not decrease the expression of LPAR1 in β-arrestin1/2-deficient HEK293 cells (Fig. S8A, B). This suggests that LPA induces LPAR1 endocytosis in a β-arrestin-dependent manner. In contrast, OMPT and amitriptyline did not induce β-arrestin-dependent endocytosis (Fig. S8B). Long-term treatment with LPA or OMPT did not decrease the protein level of FLAG-LPAR1 in HEK293 cells or LPAR1 protein in the mouse hippocampus (Fig. S8C, D). These results suggest that LPA reduces the cell surface expression of LPAR1 while retaining LPAR1 intracellularly without degrading it, consistent with previously reported findings [32]; contrarily, OMPT does not reduce cell surface LPAR1 expression. Immunoprecipitation confirmed that recruitment of endogenous β-arrestins to FLAG-LPAR1 was increased in a concentration-dependent manner with LPA treatment, but not with OMPT treatment in HEK293 cells (Fig. S8E). Overall, these results suggest that OMPT is a potent G protein-biased LPAR1 agonist.

### Continuous infusion of OMPT, but not LPA, into the hippocampus induced antidepressant-like effects

To investigate whether OMPT induces an antidepressant-like effect, OMPT was continuously infused directly into bilateral hippocampi using osmotic minipumps (Fig. 4C). Two weeks of OMPT infusion significantly decreased immobility time in the FST (Fig. 4D). OFT revealed that OMPT had no effect on distance traveled (Fig. 4E). Thus, the antidepressant-like effect of OMPT shown in the FST was not due to changes in locomotor activities. Conforming with previous reports [9, 10], infusion of LPA instead tended to increase immobility time in the FST (Fig. 4D), and decreased time spent at the center in the OFT (Fig. 4E), indicating an anxious behavior. These results suggest that long-term administration of the G protein-biased LPAR1 agonist OMPT induces antidepressant-like effects, whereas mice treated with non-biased agonist LPA may exhibit anxious behavior rather than antidepressant-like effects.

### Characterization of LPAR2–6 downstream signaling pathways using various antidepressants and LPAR agonists

We examined LPAR2–6 activities and found that most, though not all, TCAs have G protein-biased LPAR2–3 agonism; however, there was no effect on LPAR4–6 activities (Fig. S9–13). OMPT was a potent G protein-biased agonist for all LPARs (Fig. S14). Therefore, the possibility that LPARs other than LPAR1 could be involved in antidepressant effects cannot be excluded. LPAR1 was remarkably expressed in the mouse brain (Fig. S15), suggesting that LPAR1 may be a major contributor to central LPA signaling.

### Long-term administration of LPA and OMPT showed different gene expression patterns

**Fig. 5:**
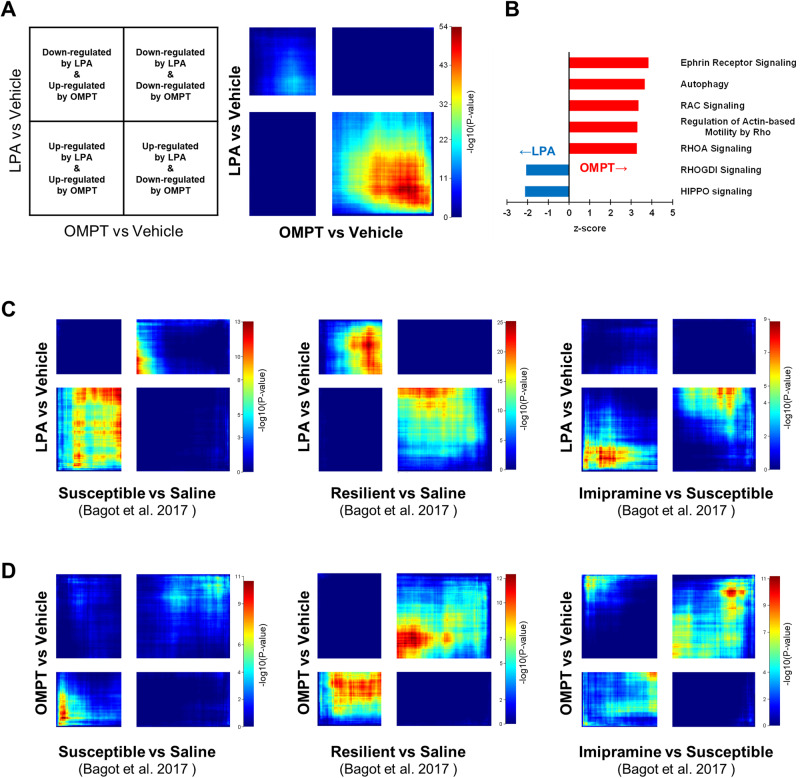
Transcriptional characterization by continuous administration of LPA or OMPT. **A** Comparison of RRHO expression patterns in the hippocampus of mice intrahippocampally treated for 2 weeks with LPA or OMPT. **B** Canonical pathways enriched with discordantly overlapping genes are presented in panel **A**. Pathways with positive z-scores indicate pathways that are predicted to be activated by OMPT. **C** Comparisons of RRHO expression patterns in the hippocampus of mice treated with LPA (Y-axis) and chronic social defeat stress models (susceptible and resilient) treated with or without imipramine (X-axis). **D** Comparisons of RRHO expression patterns in the hippocampus of mice treated with OMPT (Y-axis) and chronic social defeat stress models treated with or without imipramine (X-axis).

RNA-seq was performed to analyze gene expression changes in the hippocampus after 2 weeks infusion with LPA or OMPT. Threshold-free rank-rank hypergeometric overlap (RRHO) analysis [33, 34] was used to characterize the relationship between gene expression patterns by LPA and OMPT. RRHO heatmaps allow to visualize the pattern and significance of the overlap between two independent datasets. The datasets on each axis were ordered transcripts by differential expression *p*-values and effect size direction, and colored by -log *p*-values for overlap between ranked transcripts. RRHO revealed a substantial overlap of discordantly regulated genes between LPA and OMPT (Fig. 5A). IPA [35] using the discordantly overlapping genes revealed four of the top five canonical pathways, predicted to be activated by OMPT, to be associated with downstream signals (Rho and MAPK) of LPAR1 [36] (Fig. 5B and Fig. S16). In contrast, the pathways predicted to be activated by LPA were negatively regulated by LPAR1 [37], suggesting that long-term infusion of OMPT may activate LPAR1 signaling, whereas LPA may suppress the same.

We used RRHO analysis to compare the present RNA-seq dataset with the published RNA-seq dataset of the chronic social defeat stress model and TCA-treated mice [38]. Mice subjected to social defeat were divided into subpopulations that exhibited depression-like behaviors (susceptible) and those that did not (resilient). Depression-like behaviors in susceptible mice were ameliorated by the TCA imipramine [38]. The transcription pattern of LPA-injected mice visually showed a concordant overlap with that of susceptible mice, a discordant overlap with that of resilient mice inversely, and no directionally consistent overlap with that of imipramine-treated mice (Fig. 5C). In contrast, the transcription pattern of OMPT-injected mice seemed to show a reduced overlap with that of susceptible mice, and a concordant overlap with those of resilient and imipramine-treated mice (Fig. 5D). These results suggest that transcription pattern in the hippocampus of OMPT-treated mice may be similar to that of stress-resilient mice, while that of LPA-treated mice may be similar to that of depressed mice.

## Discussion

In the present study, we demonstrated that TCAs directly interact with LPAR1 and exhibit G protein-biased LPAR1 agonism, which may contribute to their antidepressant effects. The agonist activity of LPAR1 was found to be unique to TCAs. Previous clinical findings have suggested that TCAs may be more effective than other types of antidepressants. For example, a network meta-analysis of 21 antidepressants for the acute phase treatment of major depressive disorder showed that amitriptyline was the most effective antidepressant [1]. Another meta-analysis showed that tricyclic antidepressants are more effective than SSRIs in patients with severe depression [2]. In addition, tricyclic antidepressants were more likely to cause a switch from depression to mania in patients with bipolar depression than other classes of antidepressants [39]. The action of LPAR1 may partly explain such high responsiveness of TCAs. Our findings provide new insights into the action mechanism of TCAs and suggest that targeting LPAR1 may be a potential strategy for developing more effective antidepressants.

A typical SSRI, fluoxetine, was used in the present study as a comparison with amitriptyline. Both amitriptyline and fluoxetine acutely decreased immobility in the FST, which could be attributed to 5HT1A involvement rather than LPARs, suggesting that LPARs are not involved in the effects of acute antidepressants. In the subsequent experiments, subchronic treatment with amitriptyline significantly reduced immobility time, and this effect was blocked by Ki16425, indicating the potential involvement of LPARs in the long-term effects of amitriptyline. In contrast, two weeks of fluoxetine treatment resulted in an increase in immobility, regardless of whether it was combined with Ki16425. Previous studies have reported strain-dependent effects of long-term fluoxetine administration in the FST. For instance, treatment with fluoxetine for 12 days resulted in decreased immobility time in FST for BALB/cJ mice [40]. Conversely, studies on C57BL6/J mice reported no significant effect on immobility time with long-term fluoxetine administration [41, 42]. Moreover, Ihene et al. reported that chronic administration of fluoxetine increased immobility time in the FST [43]. In contrast, consistent reports indicate that amitriptyline reduces immobility time in the FST, irrespective of the mouse strain [44–47]. Overall, these reports and our results suggest that long-term administration of amitriptyline may exert a stronger antidepressant effect than fluoxetine in the FST, possibly through the LPARs.

While our study yielded evidence that LPAR1 signaling does not directly affect hippocampal monoamine content, previous reports have explored the potential interactions between monoaminergic signaling and LPAR signaling. For instance, the exogenous LPAR ligand, Gintonin, has been found to stimulate serotonin release from enterochromaffin cells through LPARs [48]. Additionally, TCAs, such as amitriptyline, or LPA has been shown to transactivate fibroblast growth factor receptor (FGFR) and downstream signaling in C6 glial cells [3]. Similarly, serotonin has been observed to transactivate FGFR signaling [49]. Considering these findings, it is plausible to hypothesize that TCAs, such as amitriptyline, may induce their antidepressant effects through synergistic interactions between LPAR1 and monoamine signaling pathways.

In this study, we showed that TCAs bind to LPAR1 and act as G protein-biased agonists at micromolar concentrations; notably, these concentrations are higher than therapeutic blood concentrations [50]. However, typical antidepressants accumulate in the brain, and are suggested to reach micromolar concentrations [21–23, 51, 52]. That is, antidepressants may act in the brain at concentrations more than 10 times higher than in the blood. Recently, evidence has indicated that higher concentrations of antidepressants may work in functionally meaningful ways. For example, it has been reported that the gradual increase in brain concentration of antidepressants to the micromolar levels required for interaction with low-affinity binding targets such as TrkB may be important for the onset of therapeutic effects [53]. LPAR1 may similarly be a target for TCAs at clinically meaningful concentrations.

In this study we used Ki16425, which has been reported to pharmacologically mimic the behaviors of LPAR1-deficient mice [54, 55]. Ki16425 inhibits LPAR1 (Ki value; 0.34 μM), but also inhibits LPAR2 and LPAR3 (Ki value; 6.5 μM and 0.93 μM, respectively) [56]. Moreover, some TCAs showed G protein-biased agonism in LPAR2–3 as well as LPAR1. Therefore, it is possible that LPAR2–3, in addition to LPAR1, may be involved in the antidepressant effects of TCAs. A previous review [57] and our data indicate that LPAR1 is the most abundantly expressed LPAR in the adult mouse brain. In addition, the behavioral change induced by amitriptyline in the FST was not observed in LPAR1 heterozygous mice. Therefore, the behavioral effects of amitriptyline are likely mediated, at least in part, by LPAR1 in the brain. Further clarification of the involvement of LPAR2–3 in antidepressant effects will provide vital information for the development of new antidepressants that target mechanisms other than monoamines.

We observed an interesting phenomenon in which long-term treatment with LPAR agonists LPA and OMPT, which differ in their downstream signaling bias, induced conflicting emotional behaviors and regulated distinct gene expression patterns in the hippocampus. LPA decreased the cell surface LPAR1 in a β-arrestin-dependent manner, suggesting that it may act as a functional antagonist. In contrast, G protein-biased LPAR1 agonists, OMPT and amitriptyline, did not induce β-arrestin-dependent endocytosis. Therefore, long-term administration of LPA might act as a functional antagonist and result in a behavior similar to that in LPAR1-deficient mice, whereas OMPT may be able to continue to activate LPAR1 and induce antidepressant-like effects. Thus, the concept of functional antagonism may provide a mechanism to resolve the previous inconsistent results in which LPAR1-deficient mice and LPA-treated mice both exhibited depression-like behaviors [7–10].

It has been reported that long-term intraventricular administration of LPA to normal mice with a hybrid C57BL6 x 129SvJ background did not change or decreases the immobility time of the FST [58, 59], which contradicts our results. These discrepancies may be due to differences in the site of LPA administration or differences in mouse strains. The route of administration can significantly influence the distribution and bioavailability of LPA within the brain, potentially resulting in different neural circuitry being affected. Additionally, it is worth noting that the LPA-induced increase in spontaneous behavior observed in the OFT in the Rosell-Valle et al. study may also affect the performance of the mice in the FST [59]. The changes in spontaneous behavior could potentially influence immobility levels in the FST, thereby affecting the interpretation of the antidepressant-like effects of LPA.

Recent structural studies indicated that GPCRs are highly dynamic proteins that can adopt multiple conformational states depending on their ligands [60, 61]. Different receptor conformations result in different recruitment profiles for effector proteins, such as G proteins and β-arrestins. For example, G protein-biased agonists of MOR trigger conformational changes in the intracellular loop 1 and helix 8 domains of the MOR, thereby possibly impairing β-arrestin binding or signaling [62]. The findings could allow for structure-based design of biased ligands. Recently, agonist-bound structures of human LPAR1 have been identified [63, 64]. Therefore, future analysis of the conformational states of LPAR1 induced by OMPT and TCAs may yield important information for the discovery of novel antidepressant drugs.

Our findings implied that MAPK and Rho-related signaling, which are downstream signals of LPAR1, mediate antidepressant-like effects in the hippocampus. Previous studies have reported that neuronal Rho/ROCK inhibition causes antidepressant-like effects via neuronal morphological alterations [65, 66]. Notably, LPAR1 has been reported to be heterogeneously expressed in the brain, with abundant expression in glial cells, such as oligodendrocytes and astrocytes, rather than in neurons [30]. As the Rho/ROCK pathway is important for glial cell proliferation [67], Rho signaling may play a distinct role in exhibiting antidepressant effects in LPAR1-expressing glial cells than in neurons.

In our study, mice were administered amitriptyline or CORT in their drinking water. Consequently, LPAR manipulation could potentially affect the delivery of these compounds to the brain, influencing their behavioral effects. The impact of LPAR manipulation on the brain delivery of these compounds remains a potential limitation that should be considered in the interpretation of our results and warrants further investigation in future studies.

Considering hormonal variability and comparability with many previous studies, the current study used male mice. As the prevalence and symptomatology of depression can differ between men and women, it has been suggested that there are gender differences in response to antidepressant medications [68]. The limitation of this study is that it was only addressed in male mice, and the possibility of gender-specific antidepressant effects needs to be investigated in the future.

In summary, our findings suggest that G protein-biased LPAR1 agonism may contribute to the non-monoaminergic antidepressant effects of TCAs. Further characterization of G protein-biased signaling of LPAR1 in the hippocampus can provide a basis for the development of novel antidepressants exhibiting activities other than monoamine restriction.

### Supplementary information

Supplementary information
