# Supplementary material for: G protein-biased LPAR1 agonism of prototypic antidepressants: Implication in the identification of novel therapeutic target for depression
Source: Neuropsychopharmacology. 2023 Sep 6;49(3):561–72. doi: 10.1038/s41386-023-01727-9 (PMC10789764; doi:10.1038/s41386-023-01727-9)
Supplement: Supplementary file 1 — Supplementary information [file 41386_2023_1727_MOESM1_ESM.pdf]

Supplementary information for

G protein-biased LPAR1 agonism of prototypic antidepressants: Implication in the identification of novel therapeutic target for depression

This file includes:

Supplementary materials and methods

References

Supplementary figures 1-16

Supplementary materials and methods

Preparation of tricyclic antidepressant (TCA)-immobilized magnetic beads (TCA-beads)

Nortriptyline was fixed on NHS-beads (TAS8848 N1141; Tamagawa Seiki, Nagano, Japan) as per manufacturer's instructions. To prepare different fixed levels of TCA-beads, 0.3 or 0.5 mM nortriptyline was incubated with NHS-beads. Non-fixed beads were control beads. Nortriptyline amount immobilized on beads was calculated by quantifying the N-hydroxysuccinimide, cleaved by the ligand immobilization reaction (see Supplementary Fig. 1), using high-performance liquid chromatography (outsourced to Tamagawa Seiki). The average fixation amount was 19.3 and 27.3 nmol/mg using 0.3 and 0.5 mM nortriptyline, respectively.

Affinity purification of lysophosphatidic acid receptor 1 (LPAR1) with TCA-beads

LPAR1-overexpressing membrane lysates derived from RH7777 were obtained from Chantest (#A324; Cleveland, OH, USA), and prepared from HeLa cells (RIKEN BRC, RBRC-RCB0007) transfected with FLAG-LPAR1 plasmid. HeLa cells were transfected with FLAG-tagged human LPAR1-encoding pCAGGS plasmid using ScreenFectA plus and the enhancer (SFA P-reagent; Fujifilm Wako, Osaka, Japan), as per manufacturer's instructions.

The lysates were incubated with control beads or TCA-beads for 2 h at 4 °C in binding buffer (20 mM HEPES-NaOH, pH 7.9, 100 mM NaCl, 10 mM $MgCl_2$, 10% glycerol, 0.2% NP-40) with protease inhibitor cocktail (Sigma, St.Louis, MO, USA). Magnetic separation and washing were repeated four times with wash buffer (20 mM HEPES-NaOH, pH 7.9, 100 mM NaCl, 10 mM $MgCl_2$, 10% glycerol, 0.1% NP-40). The binding proteins were eluted by adding 1× SDS sample buffer and boiled at 100 °C for 5 min. In competitive experiments, free ligands were pre-mixed with the lysate and incubated with TCA-beads. Eluted LPAR1 was detected by immunoblotting.

Immunoblotting

Immunoblotting was performed with individual antibodies: anti-EDG2 antibody (for LPAR1) (ab23698, Abcam, Cambridge, MA, USA), anti-DDDDK tag antibody (for FLAG) (PM020, MBL, Tokyo, Japan), anti-FLAG (M2) antibody (F1804, Sigma), anti-β-arrestin1/2 antibody (sc-74591, Santa Cruz, CA, USA), and anti-Na^+/K^+-ATPase antibody (#3010, Cell signaling, Danvers, MA, USA). Each antibody was diluted at an appropriate concentration with Can Get Signal Solution (Toyobo, Osaka, Japan) and treated with the blotting membrane.

Protein amount-adjusted lysate and eluted sample in bead affinity purification or immunoprecipitation were separated by SDS-polyacrylamide gel electrophoresis and

transblotted onto polyvinylidene difluoride membranes. The membranes were blocked with 5% nonfat dry milk in TBST (20 mM Tris-HCl, 150 mM NaCl, and 0.1% Tween 20; pH 7.4) for 1 h at room temperature. Thereafter, the membranes were incubated with primary antibodies overnight at 4 °C. After washing, the membranes were incubated with horseradish peroxidase-conjugated secondary antibodies for 1 h at room temperature. Chemiluminescence detection was performed using Immun-Star WesternC Kit (Bio-Rad, Hercules, CA, USA); net intensities of each signal were quantified using ChemiDoc Imaging Systems (Bio-Rad).

Electrical Impedance-based Biosensors (CellKey Assay)

The CellKey assay, a label-free cell-based assay that detects G protein activation, was performed as described previously (1, 2). C6 rat glial cells (obtained from RIKEN, RBRC-RCB2854) were used to detect G protein activation via LPARs (1). HEK293 cells stably expressing human μ-opioid receptors (MOR) were used to detect G protein activation via the MOR (2). Cells were cultured at a density of 2×10^4 cells/well for C6 cells and 5×10^4 cells/well for HEK293 cells on a standard CellKey 96-well microplate. Just before the assay, cells were washed with assay buffer (HBSS (Thermo Fisher Scientific, Waltham, MA, USA) with 20 mM HEPES and 0.1% BSA) and allowed to equilibrate in the assay buffer for 30 min

at 29 °C. The CellKey instrument applied small voltages every 10 s and measured the impedance of the cell layer (ΔZ). In this study, a 5-min baseline was recorded, drugs were added; subsequently ΔZ was measured for 10 min. The extent of changes in ΔZ was expressed in terms of ΔZ maximum after drug injection.

Transforming growth factor α (TGFα) shedding assay

The TGFα shedding assay, which measures the activation of specific GPCR-dependent G protein signaling, was performed as described previously (3), with minor modifications. HEK293FT cells (obtained from Life Technologies, cat. no. R700-07) were seeded in 60-mm culture dishes (8×10^5 cells/dish) in DMEM (Nissui Pharmaceutical, Tokyo, Japan) supplemented with 10% fetal bovine serum, glutamine, penicillin, and streptomycin (growth medium). After a 1-day culture, cells were transfected with the transfection solution that was prepared by combining 8 μL (per dish, hereafter) polyethyleneimine (PEI) solution (1 mg/mL; Polysciences, Warrington, PA, USA) and a plasmid mixture consisting of 400 ng human LPAR-encoding plasmids, 1000 ng alkaline phosphatase (AP)-tagged TGFα (AP-TGFα)-encoding plasmid with or without 200 ng chimeric Gα subunit protein ($G\alpha_{q/i1}$ for LPAR1, LPAR5, and LPAR6, and $G\alpha_{q/s}$ for LPAR2 and LPAR4)-encoding plasmid in 400 μL of Opti-MEM (Thermo Fisher Scientific). After a 1-day culture, the transfected cells were harvested

by trypsinization, neutralized with the growth medium, and collected by centrifugation at room temperature at 200 g for 5 min. Cells were suspended in HBSS containing 5 mM HEPES (pH 7.4) and were left for 10 min to remove extra AP-TGFα released during trypsinization. After centrifugation, cells were resuspended in 12 mL of HEPES-HBSS and seeded in a 96-well plate (90 μL/well). Cells were incubated at 37 °C for 30 min to allow the cells to attach. Test compounds were diluted in 0.01% fatty-acid-free grade BSA (Fujifilm Wako)-containing HEPES-HBSS (assay buffer) and added to the cells (10 μL/well). For LPAR4–6, 10 μM Ki16425, an LPAR1–3 antagonist, was pretreated 5 min before the addition of test compounds. After a 1-h incubation, cells in 96-well plates were centrifuged, and the conditioned medium (80 μL) was transferred to an empty 96-well plate. The AP reaction solution (a mixture of 10 mM p-nitrophenyl phosphate, 120 mM Tris-HCl (pH 9.5), 40 mM NaCl, and 10 mM $MgCl_2$) was added to plates containing cells and the conditioned medium (80 μL/well). Absorbance at a wavelength of 405 nm was measured using a microplate reader (Varioskan LUX multimode microplate reader, Thermo Fisher Scientific) before and after 1- or 2-h incubation at room temperature. All values were calculated by subtracting the background activity induced by compounds in empty plasmid-expressing HEK293FT cells. The AP-TGFα release percentages were fitted to a four-parameter sigmoidal concentration-response curve, using the GraphPad Prism 8 software (v8.4.3, GraphPad Software, San

Diego, CA, USA), and the EC_{50} and E_{max} values were obtained therefrom. E_{max}/EC_{50} values of compounds were normalized by the E_{max}/EC_{50} value of LPA to calculate RAi (4), which was then base-10 log-transformed (LogRAi) and used as the potency of signaling activation.

NanoBiT-based β-arrestin1 recruitment assay

The NanoBiT PPI assay-based β-arrestin1 recruitment assay was performed as described previously (5), with minor modifications. Human full-length β-arrestin1 was N-terminally fused to a large fragment (LgBiT; forming LgBiT- βarr1) of NanoBiT luciferase with a 15-amino-acid flexible linker (GGSGGGGSGGSSSGG). Human LPAR1–6 were C-terminally fused to a small fragment (SmBiT; forming LPARs-SmBiT) with the 15-amino-acid flexible linker. The LgBiT- βarr1 and the LPARs-SmBiT constructs were inserted into a pCAGGS expression plasmid vector. Transfection into HEK 293FT cells was performed as described in the TGFα shedding assay using the PEI method (200 ng LgBiT- βarr1, 1000 ng LPAR-SmBiT, and 8 μL of 1 mg/mL PEI solution per dish). After a 1-day culture, transfected cells were collected with 0.5 mM EDTA-containing PBS, centrifuged, and suspended in 2 mL of assay buffer. The cell suspension was seeded in a white 96-well plate at a concentration of 5×10^5 cells/mL (80 μL/well) and loaded with 20 μL of 50 μM coelenterazine (Cayman, Ann Arbor, MI, USA) diluted in the assay buffer. After 2-h incubation at room temperature, the plate was measured

(0.5 s/well) for baseline luminescence (Varioskan LUX multimode microplate reader) and 20 μL of test compounds diluted in the assay buffer were manually added. The plate was read 30 times (0.18 s/well) at room temperature. The luminescent signal was normalized to initial count, and fold-change values over 5–10 min after test compound stimulation were averaged. The fold-change β-arrestin recruitment signals were fitted to a four-parameter sigmoidal concentration-response, and the EC_{50}, E_{max}, and LogRAi values were obtained as described above.

Compounds used in GPCR assays

Amitriptyline (A0908), imipramine (I0971), nortriptyline (N0957), mianserin (M2623), mirtazapine (M2151), maprotiline (M2527), duloxetine (D4223), milnacipran (M2133), venlafaxine (V0110), paroxetine (P1977), fluoxetine (F0750), citalopram (C2370), and fluvoxamine (F0858) were obtained from Tokyo chemical industry (Tokyo, Japan). Clomipramine (C7291-1G) and desipramine (D3900-1G) were obtained from Sigma. Vortioxetine (23694), anandamide phosphate (pAEA, 10180), and 1-oleoyl-2-methyl-sn-glycero-3-phosphothionate (OMPT, 10005707) were obtained from Cayman. Trazodone (093114) was obtained from Fujifilm Wako. 1-oleoyl-2-hydroxy-sn-glycero-3-phosphate (LPA, 857130) and VPC31143(R) (857353P) was obtained from Avanti polar lipids (Alabaster, AL,

USA). (R, S)-ketamine was provided by Professor Kenji Hashimoto, Chiba University (Japan).

Behavioral procedures

The forced swim test (FST) was performed in a clear acrylic cylinder (20 cm diameter), which was filled up to 16 cm with water (23–25 °C). Mice were placed in the cylinder and left there for 7 min. The entire session was videotaped from the side of the cylinder, and immobility time was measured by the SMART video tracking system (SMART v3.0.06, Panlab, Barcelona, Spain). Immobility time was defined as the time that global activity was less than or equal to 30.0 cm^2/s. The last 5 min of immobility time during the test period was used to evaluate the FST.

The open field test (OFT) was performed in a square chamber (36 × 36 × 30 cm) made of white polyvinyl chloride. Mice were placed around the novel open-field chamber and allowed to explore for 15 min. The entire session was videotaped. The time spent in the center of the chamber (18 × 18 cm) and the distance traveled were measured using the SMART video tracking system.

For the sucrose preference test (SPT), mice were exposed to drinking water substituted with 2% sucrose for 3 nights to avoid neophobia. The day before the SPT, mice were housed individually and subjected to water deprivation. Two pre-weighed bottles (one containing tap

water and another containing 2% sucrose solution) were presented to each mouse for 4 h. The positions of water and sucrose bottles were switched 0.5 and 2 h after the start of the SPT to avoid any place preference. The bottles were weighed again, and the difference in weight between 0.5–4 h was used to calculate the volume intake from each bottle. Sucrose preference was expressed as the percentage of sucrose intake relative to the total intake.

Quantification of hippocampal monoamines using HPLC

The content of monoamines in the hippocampi of mice was assessed after subchronic treatment with amitriptyline (160 mg/L, two weeks). Mice were anesthetized and quickly decapitated, and the collected hippocampi were immediately frozen in liquid nitrogen and stored at -80°C until use. The extraction and analysis of serotonin and noradrenaline content in the hippocampi were outsourced to Arcrize Japan Co., Ltd. (Fukuoka, Japan). Briefly, frozen hippocampi were homogenized with 0.2 M perchloric acid (including 100 μM EDTA-2Na) and cooled on ice for 30 min, followed by centrifugation at 12,000×g for 5 min. A 50 μL aliquot of the supernatant was mixed with 10 μL of 1 M CH_3COONa. Finally, ultrafiltration with a 0.2 μm filter was conducted at 12,000×g for 2 min, and the samples were analyzed by ion-exchange HPLC with electrochemical detection. The potential of working electrode was +450 mV. The separation HPLC column was cation exchange column (0.2 mm I.D. ×200

mm). The composition of mobile phase was mixture of 0.1 M phosphate buffer (pH 6.0) and methanol (70:30, v/v) containing 30 mM potassium and 50 mg/L EDTA-2Na. The flow-rate was 250 μL/min.

Flow cytometry-based LPAR1 endocytosis assay

HEK293A cells (obtained from Thermo Fisher Scientific, cat. no. R705-07) and HEK293A cells lacking β-arrestin1/2 were used in this assay. Cells were transfected with FLAG-tagged human LPAR1-encoding pCAGGS plasmid using ScreenFectA plus and the enhancer (Fujifilm Wako). Cell suspension and plasmid DNA-lipid complex were mixed and transfected to 12-well plates (8×10^5 cells/well). After 24-h culture, the medium was changed to the serum-starved growth medium and incubated for an additional 24 h at 37 °C. Then, cells were treated with test compounds for 15, 30, or 60 min at 37 °C. The reaction was stopped with ice-cold PBS with 1 mM EDTA. Cells were harvested and treated with anti-FLAG M2-FITC antibody (Sigma) with blocking reagent (Human BD Fc Block; BD Biosciences, Franklin Lakes, NJ, USA) on ice for 20 min to label FLAG-LPAR1 remaining on the cell surface. IgG-FITC isotype control (Sigma) was used as a non-staining negative control. After washing with ice-cold PBS with 0.5% BSA and protease inhibitor cocktail, cells were resuspended with PBS. Flow cytometry was performed by using CytoFLEX (Beckman Coulter, Brea, CA, USA).

Quantification of LPAR1 by liquid chromatography-tandem mass spectrometry (LC-MS/MS)

Membrane fraction from mice hippocampi was prepared with a Plasma Membrane Protein Extraction Kit (BioVision, Milpitas, CA, USA) according to the manufacturer's instructions. Briefly, bilateral hippocampi were minced with a razor blade. The samples were homogenized in 1.0 mL of cold homogenization buffer containing protease inhibitor cocktail using Dounce homogenizer by 30 strokes with loose pestle and then by 20 strokes with a tight pestle. After transferring homogenate to a new tube, differential centrifugation was conducted at 4 °C, for 10 min at 700×g, and 30 min at 10,000×g. After the 10,000×g centrifugation, the supernatant was collected as the cytosol fraction. The residual pellet (crude membrane fraction) was resuspended with 200 μL of Upper Phase Solution. Then, 200 μL of Lower Phase Solution was added, and the sample was vortexed. After centrifugation at 1,000×g for 5 min at 4 °C, the upper phase sample was transferred to a new 1.5-mL tube, and this process was repeated. The upper phase samples from these steps were combined and diluted with 1.5 mL of water and kept on ice for 5 min. Samples were centrifuged at 20,000×g, for 30 min at 4 °C, pellet (plasma membrane fraction) was re-suspended with 50 μL of 1×PTS buffer (100 mM Tris-HCl (pH 9.0), 12 mM sodium lauroylsarcosinate, 12 mM sodium deoxycholate) and

sonicated on ice for 15 min. The samples were heated at 95 °C for 5 min, and protein concentrations were analyzed using BCA protein assay. The protein amount adjusted membrane fraction (10 μg/10 μL in 1×PTS buffer) was treated with 1 μL of 100 mM (+/-) dithiothereitol (Fujifilm Wako) in 50 mM ammonium bicarbonate (AmBic, Fujifilm Wako) for 30 min, then treated with 1 μL of 550 mM iodoacetamide in 50 mM AmBic for 30 min at room temperature in the dark. Next, samples were incubated with 0.5 μg of lysyl endopeptidase (Fujifilm Wako) for 3 h at room temperature followed by 0.5 μg of trypsin (Promega, Madison, WI, USA) overnight at 37 °C. After trypsin digestion, 54 μL of ethyl acetate (Fujifilm Wako) and 0.54 μL trifluoroacetic acid (Fujifilm Wako) were added and vortexed for 2 min. Samples were then centrifuged at 15600×g for 2 min at 25 °C. After discarding the upper phase sample as rigorously as possible, the lower phase sample was evaporated at 37 °C for 1 h by using a centrifugal evaporator (CVE-3100, EYELA, Tokyo, Japan). After drying by vacuum centrifugation, the digested samples were dissolved in 5% acetonitrile/0.1% trifluoroacetic acid and stable isotope labeled internal standard peptides, which have the same amino acid sequence as the target peptide, were spiked at a fixed amount. The peptide samples were desalted using GL-Tip SDB and GC (GL Sciences, Tokyo, Japan), and reconstituted with 0.1% trifluoroacetic acid. Each sample (1 μg) was subjected to LC-MS/MS. The samples were analyzed by parallel reaction monitoring mode on TripleTOF6600 (SCIEX, Framingham,

MA, USA) interfaced with a nanoLC400 (SCIEX). The target peptide for LPAR1 is LTVSTWLLR, and m/z values for precursor ions are 544.8 and 549.8 for unlabeled and labeled peptides, respectively. The target peptides for Na^+/K^+-ATPase are AAVPDAVGK and VDNSSLTGESEPQTR. m/z values for precursor ions of AAVPDAVGK are 414.2 and 419.2 for unlabeled and labeled peptides, respectively, and those of VDNSSLTGESEPQTR are 810.4 and 815.4, respectively. The LC-MS/MS data was analyzed using Skyline. The chromatograms of LTVSTWLLR were extracted by y6, y7, and y8 product ions. Those of AAVPDAVGK were extracted by y4, y6, and y7 ions, and those of VDNSSLTGESEPQTR were extracted by y4, y6, y8, and y9 ions. The abundance of each peptide was calculated as target-to-internal standard peak area ratio. The expression levels of LPAR1 were normalized by those of Na^+/K^+-ATPase.

Immunoprecipitation

HEK293A cells were transfected with FLAG-tagged human LPAR1-encoding pCAGGS plasmid by the PEI method (0.5 μg plasmid and 2 μL of 1 mg/mL PEI solution per well in 12-well plates). After 6-h incubation, the medium was changed to the serum-starved growth medium and incubated for an additional 18 h at 37 °C. Then, cells were treated with test compounds for 10 min at 37 °C and collected in 200 μL/well of IP lysis buffer (25 mM Tris-

HCl (pH 7.4), 150 mM NaCl, 1% NP-40, 1 mM EDTA, 5% glycerol) with protease inhibitor cocktail (Nacalai, Kyoto, Japan). Forty µL of anti-FLAG M2 monoclonal antibody-coupled magnetic beads (Sigma) were added to the lysate and incubated for 2 h at 4 °C. The bead-bound proteins were eluted with 40 µL of elution buffer (100 mM glycine-HCl (pH 2.5), 150 mM NaCl), and then neutralized with 4 µL of 1 M Tris-HCl (pH 8.0). Lysates before and after immunoprecipitation were used for immunoblotting as described above.

Quantification of LPARs mRNA

Total RNA was isolated from mouse whole brain or the hippocampus. Tissues were homogenized in 1% β-mercaptoethanol in RLT buffer using a TissueLyser II (Qiagen, Hilden, Germany) with a stainless-steel bead. The homogenates were centrifuged, and the supernatant was collected and added with the same volume of 70% ethanol. Subsequent total RNA isolation was performed using the RNeasy mini kit (Qiagen) following the manufacturer's instructions. The cDNA was obtained from total RNA using PrimeScript RT Master Mix (Takara bioscience, Shiga, Japan). Real-time PCR was performed with the Thermal Cycler Dice Real-Time System II (Takara bioscience). For detection of GAPDH and LPAR1–6, specific primers and SYBER Premix Ex Taq II (Takara bioscience) were used. Forward and reverse sequences of the primers are as follows.

GAPDH: 5'-aggtcggtgtgaacggatttg-3' (forward), 5'-tgtagaccatgtagttgaggtca-3' (reverse)

LPAR1: 5'-gaggaatcgggacaccatgat-3' (forward), 5'-acatccagcaataacaagaccaatc-3' (reverse)

LPAR2: 5'-cattctgggggcatttgtgg-3' (forward), 5'-ttgaccagtgagttggcctc-3' (reverse)

LPAR3: 5'-gtcttaggcgccttcgtgg-3' (forward), 5'-ttgcacgttacactgcttgc-3' (reverse)

LPAR4: 5'-gcgagttgccagtttacacg-3' (forward), 5'-ttgagtgcccaagaaagagtgt-3' (reverse)

LPAR5: 5'-gtctccctctcaacgctctg-3' (forward), 5'-ggggaagtgacagggtgaag-3' (reverse)

LPAR6: 5'-ctgtaagctgcactgcctga-3' (forward), 5'- atgctgaacatgcacccgta-3' (reverse)

PCR products were confirmed to be the target products by agarose gel electrophoresis based on size compared to markers. To obtain a standard curve, we cut gels of target bands, and purified each PCR product using NucleoSpin Gel and PCR Clean-up kit (Takara Bioscience). The copy number was determined from the concentration and bp of the purified PCR product. Two-fold serial dilutions of each PCR product were made and used as templates to generate standard curves for real-time PCR. Copy numbers of LPARs and GAPDH in cDNA generated from whole brain and hippocampus were calculated from the standard curve.

RNA-seq and data analysis

To limit the contribution of acute effects of behavioral testing on gene expression, mice were sacrificed 2 days after the final behavioral test. Whole hippocampi were rapidly isolated and

frozen in liquid nitrogen. Total RNA was extracted using the AllPrep DNA/RNA mini kit (Qiagen) with DNase I to eliminate any contaminating DNA. The RNA integrity number values of all samples were above 8.0. Library preparation and RNA-seq were outsourced to Macrogen Japan (Kyoto, Japan). Libraries were prepared using the TruSeq Stranded mRNA library prep kit (Illumina, San Diego, CA, USA), and were sequenced on the NovaSeq 6000 (Illumina) with 100 bp paired-end reads.

Raw reads were trimmed for adaptor sequences and low-quality reads using Trimmomatic (v0.39) with the following parameters: LEADING, 20; TRAILING, 20; CROP, 100; MINLEN, 20. Trimmed reads were then aligned to the mm10 reference genome using STAR (v2.7.9a) with default parameters. Uniquely mapped reads were used to obtain the estimated read count and transcripts per million (TPM) using RSEM (v1.3.3). Estimated read count was processed using TCC-GUI (6) with default parameters. TCC-GUI uses the TMM normalization method, and edgeR was used to determine the differentially expressed genes. Before processing with TCC-GUI, poorly expressed genes were filtered by removing those with an average TPM value of 0 and those with an average TPM value in the bottom 25% of the remaining genes.

To evaluate the overlap of expression patterns between two ranked gene lists, threshold-free genome-wide transcriptomic overlap analysis was conducted using rank-rank

hypergeometric overlap (RRHO2, v1.0). Transcripts from each TCC-GUI comparison were ranked by -log10 (p-value) multiplied by the sign of fold change. The ranked gene lists were used to generate matrices of overlapped genes between differential expression lists of interest. Discordantly overlapped genes between LPA and OMPT-elicited transcriptional patterns were extracted from the data analyzed by RRHO2. Using the gene lists, canonical pathways were generated using Ingenuity Pathway Analysis (IPA, Release Date: 2021-10-22, Qiagen).

A publicly available RNA-seq dataset (Gene Expression Omnibus database: GSE81672) was obtained for comparison with our RNA-seq data. The data were processed using the pipeline described above and analyzed by RRHO2.

References

1. Kajitani N, Miyano K, Okada-Tsuchioka M, Abe H, Itagaki K, Hisaoka-Nakashima K, et al. (2016): Identification of Lysophosphatidic Acid Receptor 1 in Astroglial Cells as a Target for Glial Cell Line-derived Neurotrophic Factor Expression Induced by Antidepressants. *J Biol Chem*. 291:27364-27370.

2. Hisaoka-Nakashima K, Miyano K, Matsumoto C, Kajitani N, Abe H, Okada-Tsuchioka M, et al. (2015): Tricyclic Antidepressant Amitriptyline-induced Glial Cell Line-derived Neurotrophic Factor Production Involves Pertussis Toxin-sensitive G α i/o Activation in Astroglial Cells. *J Biol Chem*. 290:13678-13691.

3. Inoue A, Ishiguro J, Kitamura H, Arima N, Okutani M, Shuto A, et al. (2012): TGF α shedding assay: an accurate and versatile method for detecting GPCR activation. *Nat Methods*. 9:1021-1029.

4. Ehlert FJ, Griffin MT, Sawyer GW, Bailon R (1999): A simple method for estimation of agonist activity at receptor subtypes: comparison of native and cloned M3 muscarinic receptors in guinea pig ileum and transfected cells. *J Pharmacol Exp Ther*. 289:981-992.

5. Shihoya W, Izume T, Inoue A, Yamashita K, Kadji FMN, Hirata K, et al. (2018): Crystal structures of human ET_B receptor provide mechanistic insight into receptor activation and partial activation. *Nat Commun*. 9:4711.

6. Su W, Sun J, Shimizu K, Kadota K (2019): TCC-GUI: a Shiny-based application for differential expression analysis of RNA-Seq count data. *BMC Res Notes*. 12:133.

Supplementary figures

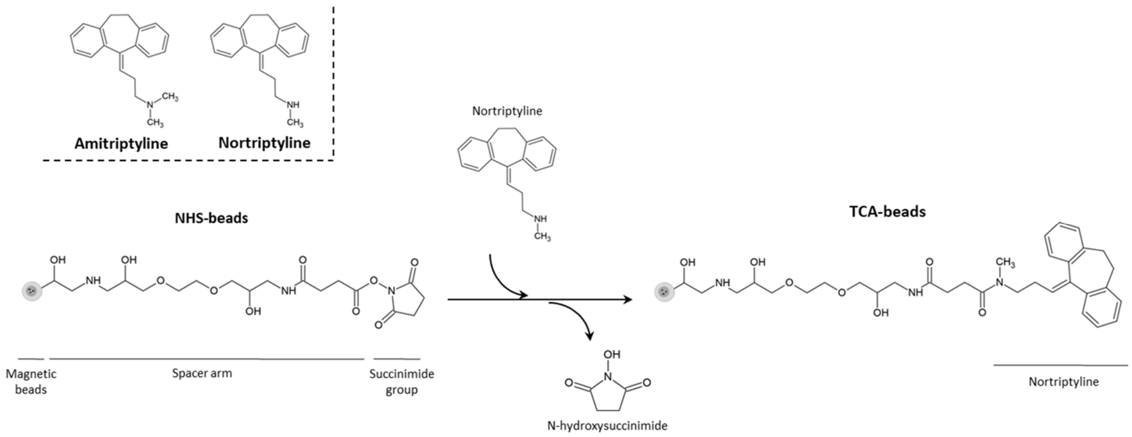

Fig. S1. Synthesis of tricyclic antidepressant (TCA)-beads.

The secondary amine of nortriptyline reacts with the succinimide group of NHS-beads. As a result, nortriptyline covalently attaches on the beads. The structure of nortriptyline is the same as amitriptyline except for the secondary amine.

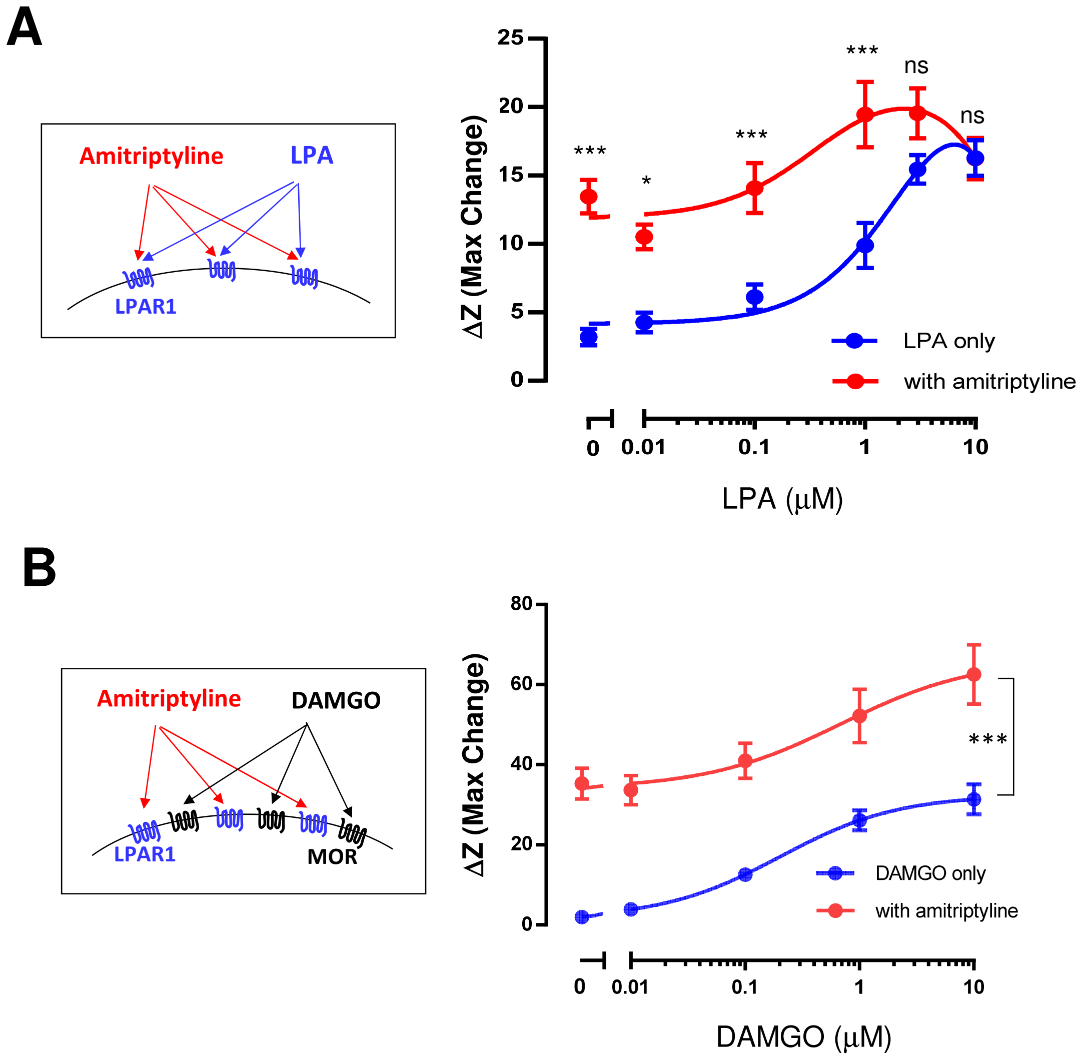

Fig. S2. Combined effects of amitriptyline (Ami) on the increase in ΔZ by GPCR agonists.

(**A**) C6 cells are treated with vehicle or LPA (0.01, 0.1, 1, 3, and 10 μM) with or without Ami (25 μM) for 10 min. The extents of changes in ΔZ is presented as the maximum ΔZ after the injection of each ligand. N=10–12. Data are represented as the mean ± SEM. Statistical significance was calculated using two-way ANOVA (the LPA concentration: $P < 0.0001$, the amitriptyline combination: $P < 0.0001$, the interaction effects: P=0.005.) with Sidak's multiple

comparisons test (***$P < 0.001$, *$P < 0.05$, ns: not significant, versus the same dose of LPA only). (**B**) MOR-HEK cells are treated with vehicle or DAMGO (0.01, 0.1, 1, and 10 μM) with or without amitriptyline (25 μM) for 10 min. The extents of changes in ΔZ is presented as the maximum ΔZ after the injection of each ligand. N=8. Data are represented as the mean ± SEM. Statistical significance was calculated using two-way ANOVA (the DAMGO concentration: $P < 0.001$, the amitriptyline combination: ***$P < 0.001$, the interaction effects: P=0.925).

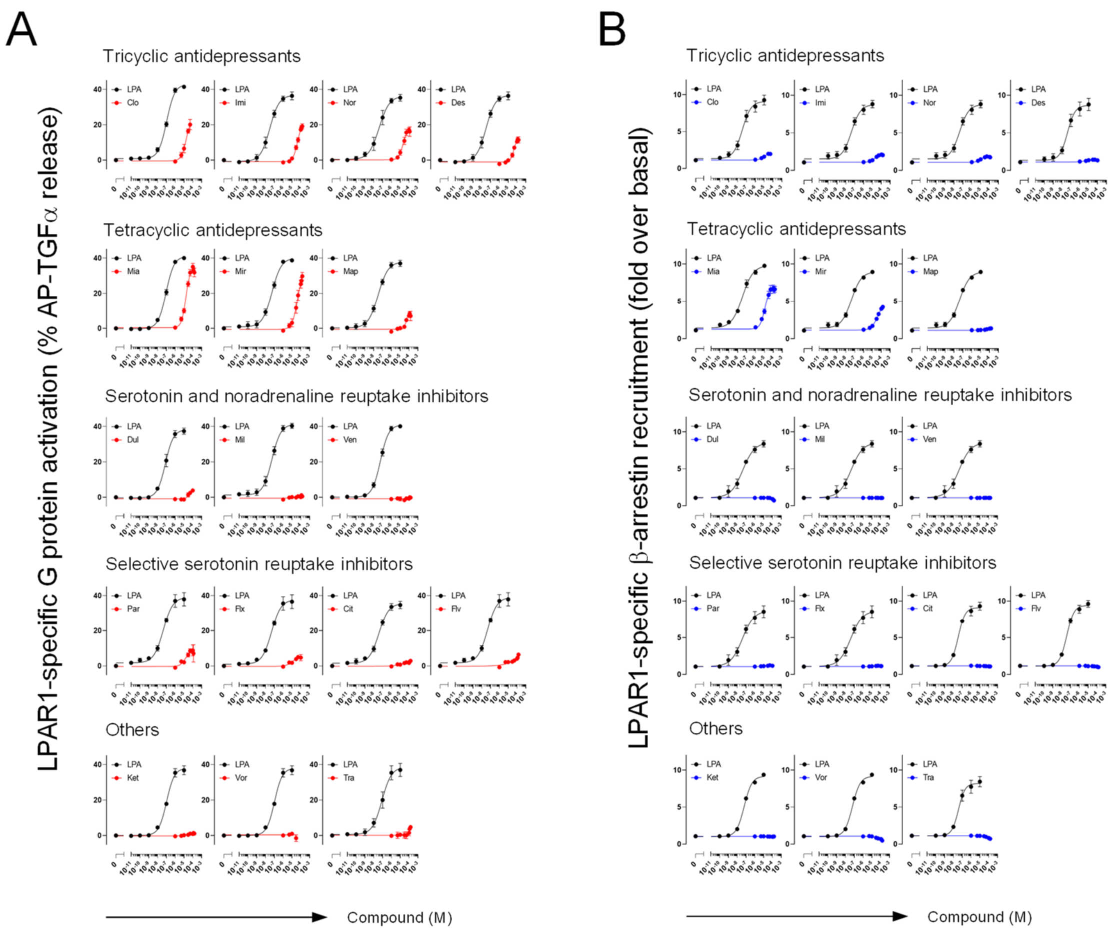

Fig. S3. Effects of various types of antidepressants on the LPAR1-specific G protein and β-arrestin signaling.

(**A**) Dose-response curves of LPA and antidepressants, including clomipramine (Clo), imipramine (Imi), nortriptyline (Nor), desipramine (Des), mianserin (Mia), mirtazapine (Mir), maprotiline (Map), duloxetine (Dul), milnacipran (Mil), venlafaxine (Ven), paroxetine (Par), fluoxetine (Flx), citalopram (Cit), fluvoxamine (Flv), ketamine (Ket), vortioxetine (Vor), and trazodone (Tra), for the LPAR1-specific G protein activation and (**B**) β-arrestin recruitment.

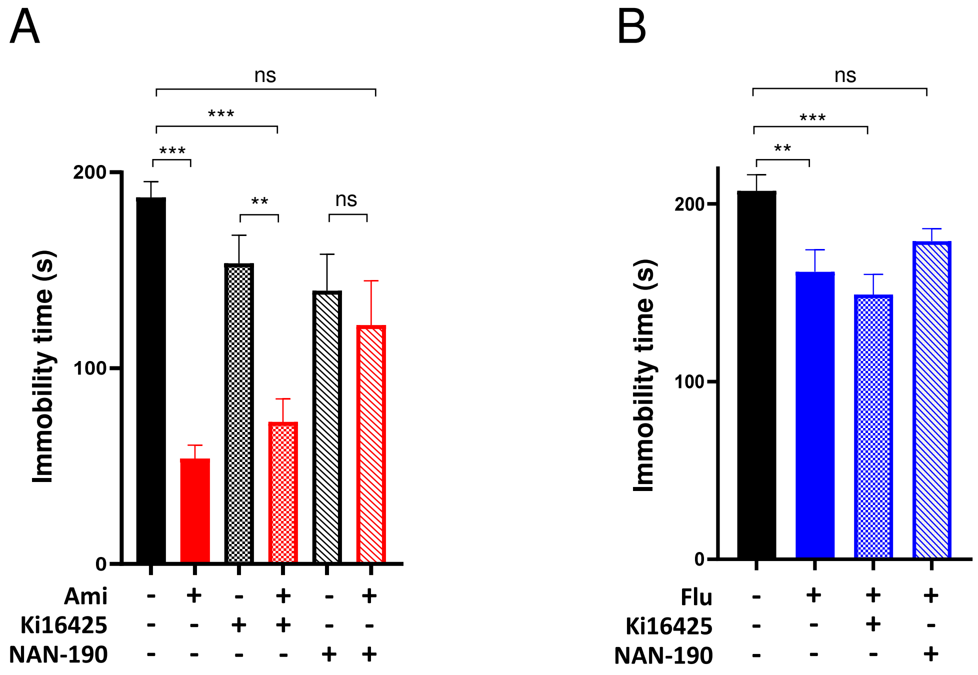

Fig. S4. Effects of various antagonists on the acute effects of antidepressants in the forced swim test (FST).

(**A**) Mice were treated with Ki16425 (LPAR1–3 antagonist; 10 mg/kg, i.p.), NAN190 (5HT1A antagonist; 0.5 mg/kg, i.p.), or vehicle. Amitriptyline (Ami; 10 mg/kg, i.p) was administered 30 min after antagonist treatment, and the FST was performed 30 min after Ami administration. N=9–16. Data are presented as means ± SEM. Statistical significance was calculated using the Kruskal–Wallis test with Dunn's multiple comparisons test (**P < 0.01, ***P < 0.001, ns: not significant). (**B**) Mice were treated with Ki16425, NAN190, or vehicle. Fluoxetine (Flu; 10 mg/kg, i.p.) was administered 30 min after antagonist treatment, and the FST was performed 30 min after Flu administration. N=9. Statistical significance was calculated using one-way ANOVA with Sidak's multiple comparisons test (**P < 0.01, ***P < 0.001, ns: not significant).

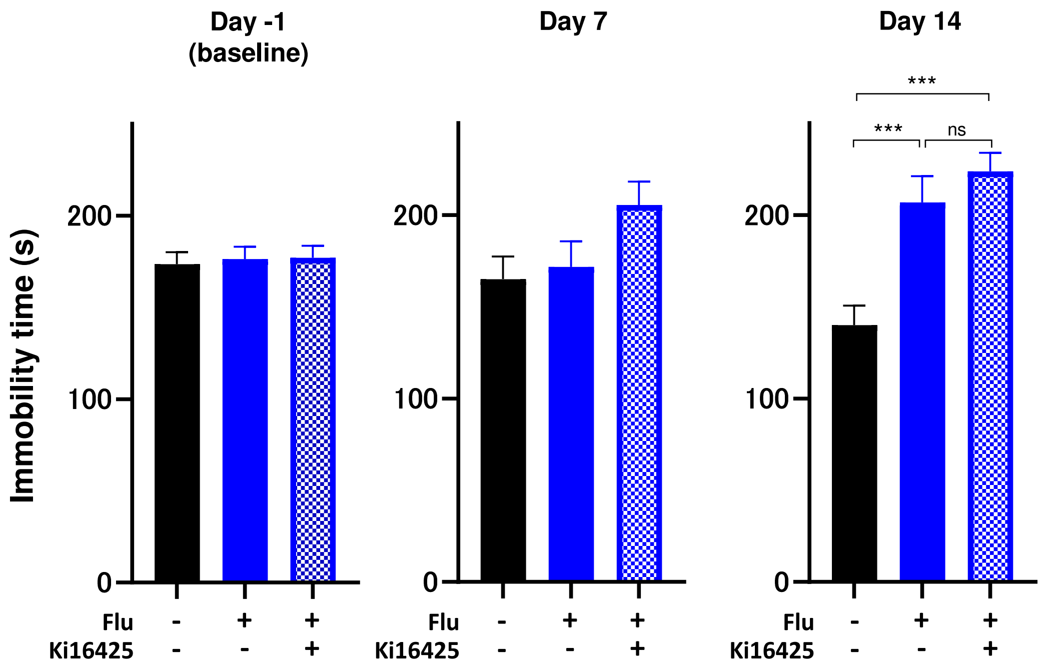

Fig. S5. Effects of subchronic treatment with fluoxetine (Flu) in combination with LPAR antagonist in the forced swim test (FST).

Mice were treated with Flu (160 mg/L), added to their drinking water, and were injected daily with Ki16425 (10 mg/kg/day, i.p.) or vehicle for 14 days. Repeated FST was performed on Day -1, 7, and 14. N=24. Data are presented as means ± SEM. Statistical significance was calculated using one-way ANOVA with Tukey's multiple comparisons test (***P < 0.001, ns: not significant).

A

Parental genotype	Offspring genotype +/+	+/-	-/-	Expected -/-
+/- × +/-	124	223	15	124
(female:male)	(65:59)	(113:110)	(6:9)	
+/- × -/-	0	18	6	18
(female:male)	(0:0)	(9:9)	(2:4)	

B

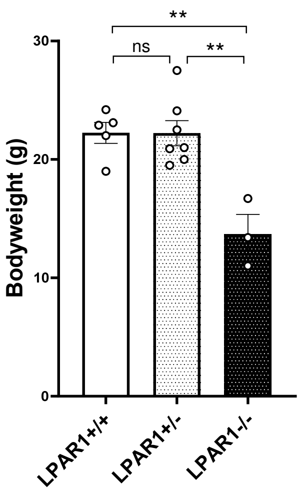

Fig. S6. Characterization of LPAR1 heterozygous mice.

(**A**) Number of genotyped offspring. The number of mice that survived for at least 6 weeks after birth was counted. The number of LPAR1-/- mice was much smaller than expected by Mendelian laws of inheritance. (**B**) Bodyweight of each genotyped mouse at the age of 6 weeks. Data are presented as the means ± SEM. Statistical significance was calculated using one-way ANOVA with Tukey's multiple comparisons test (**$P < 0.01$, ns: not significant).

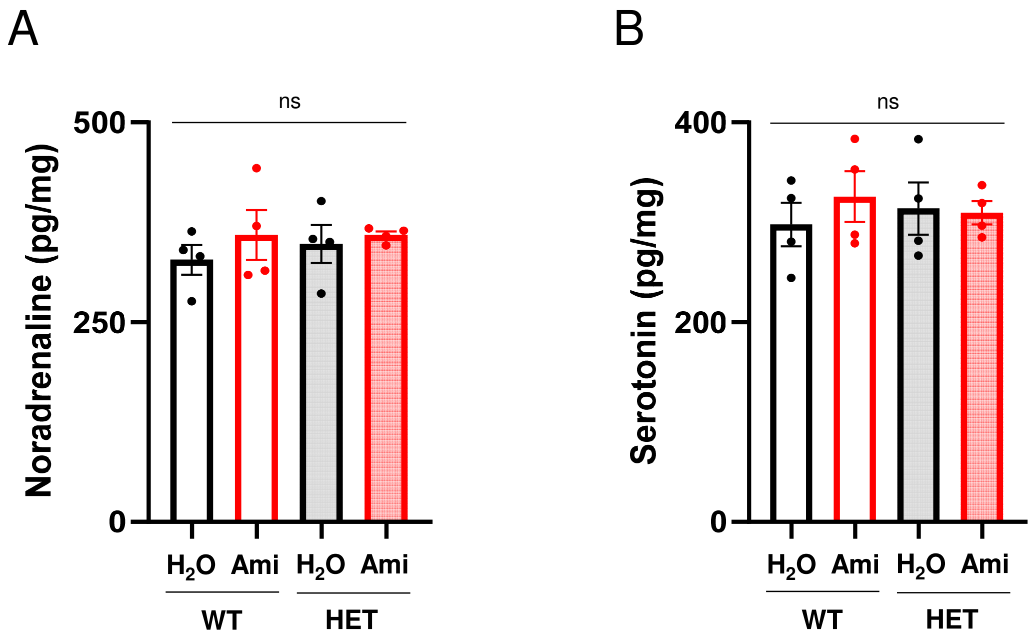

Fig. S7. Hippocampal monoamine levels in LPAR1 heterozygous mice.

(**A**) Noradrenaline content and (**B**) Serotonin content in mice hippocampus. Wild type (WT) and LPAR1 heterozygous (HET) mice were treated with Ami (160 mg/L) in drinking water for 14 days. Hippocampal noradrenaline and serotonin levels were determined by HPLC. Data are presented in amounts per hippocampal tissue weight (pg/mg). ns: not significant (one-way ANOVA).

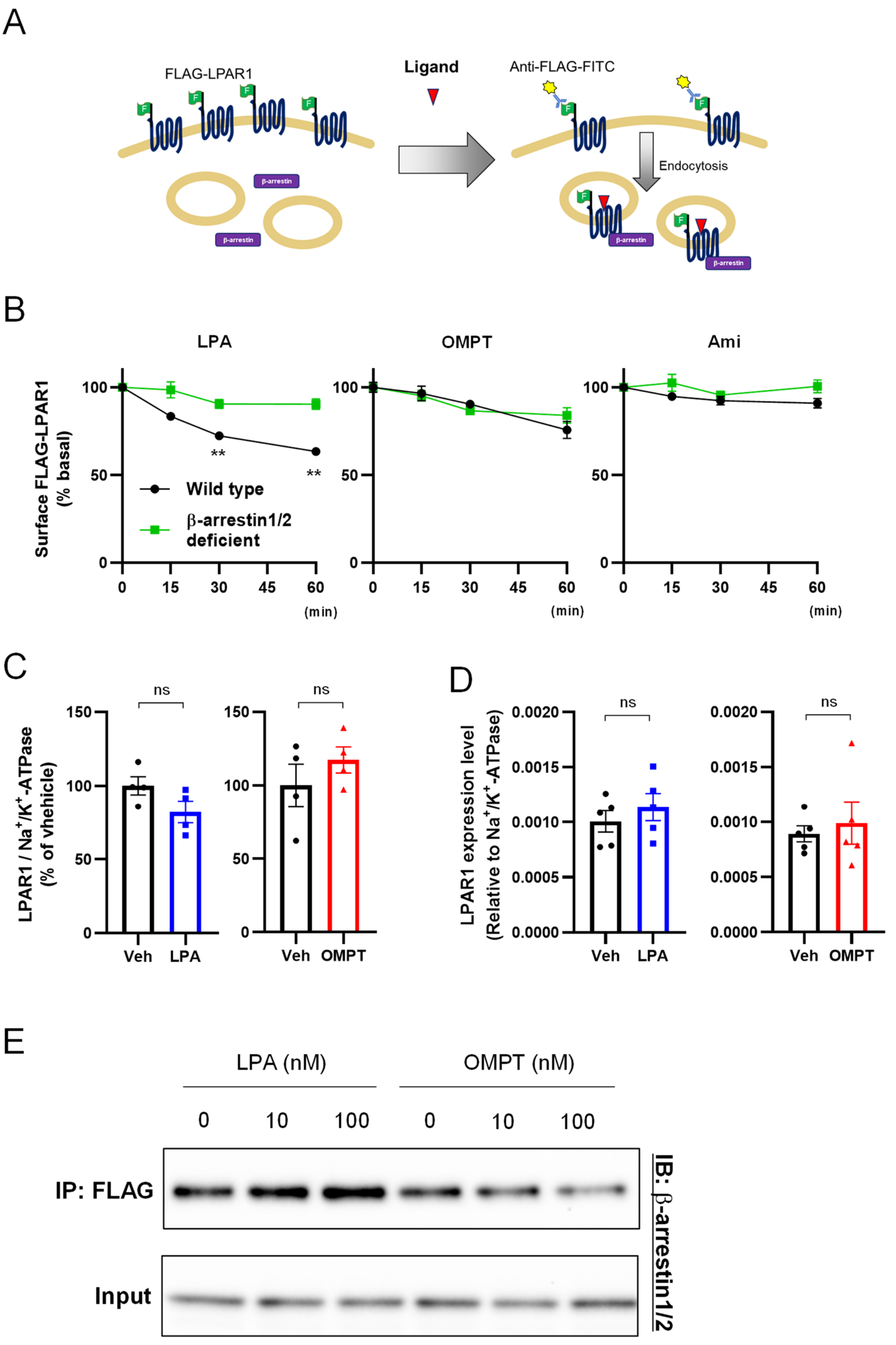

Fig. S8. Characterization of LPAR1 signaling by LPA and OMPT treatments.

(**A**) Scheme of flow cytometry-based LPAR1 endocytosis assay. (**B**) Effects of LPA, OMPT, or amitriptyline (Ami) on LPAR1 endocytosis. Cell surface expression of FLAG-tagged LPAR1 transfected in HEK293 cells (wild type) and β-arrestin 1/2 deficient cells were monitored by flow cytometry following incubation with 10 μM LPA, 10 μM OMPT, or 50 μM Ami for the indicated time (min). Data are presented as the means ± SEM. Statistical significance was calculated using Mixed-effects model with Sidak's multiple comparisons test (** $P < 0.01$). (**C**) FLAG-tagged LPAR1-transfected HEK293 cells were treated with LPA (10 μM) or OMPT (10 μM) for 24 h. FLAG-LPAR1 protein levels were determined by immunoblotting of FLAG. (**D**) LPA or OMPT was infused into mouse hippocampi for 2 weeks using osmotic pumps (concentration in pump: 15 nM, delivery rate: 0.11 μL/h). Hippocampal LPAR1 protein levels were determined by LC-MS/MS. Data are presented as a ratio of LPAR1 per Na^+/K^+-ATPase (mol/mol). ns: not significant (Unpaired t-test). (**E**) Effects of LPA or OMPT on the interaction between LPAR1 and β-arrestins. Immunoblots of β-arrestin1/2 from lysates before (Input) or after immunoprecipitation with a FLAG-specific antibody (IP: FLAG). Lysates were obtained from FLAG-tagged LPAR1-transfected HEK293 cells treated with indicated doses of LPA or OMPT for 10 min.

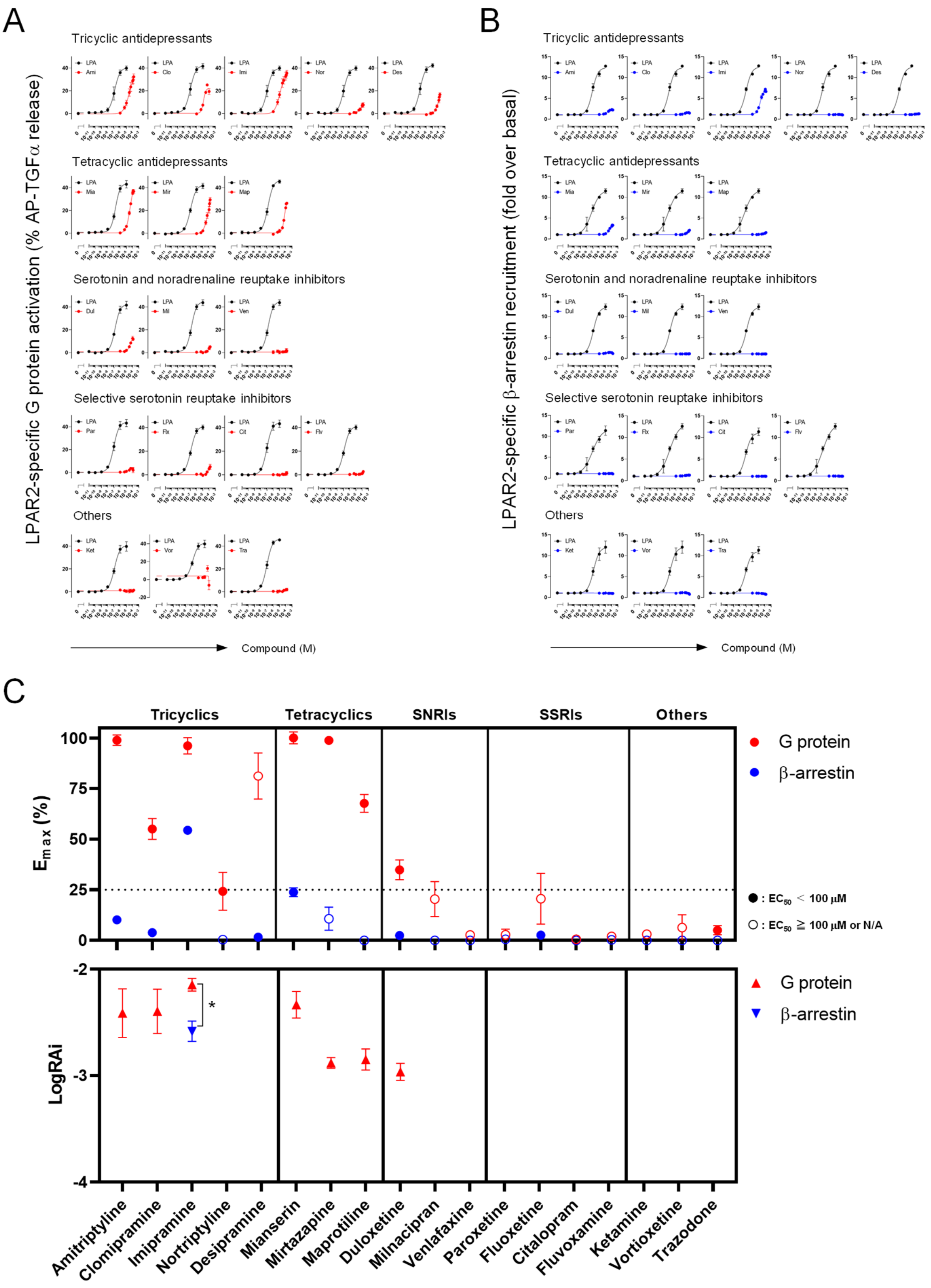

Fig. S9. Effects of various types of antidepressants on LPAR2-specific G protein and β-arrestin signaling.

(**A**) Dose-response curves of LPA and antidepressants, including amitriptyline (Ami), clomipramine (Clo), imipramine (Imi), nortriptyline (Nor), desipramine (Des), mianserin (Mia), mirtazapine (Mir), maprotiline (Map), duloxetine (Dul), milnacipran (Mil), venlafaxine (Ven), paroxetine (Par), fluoxetine (Flx), citalopram (Cit), fluvoxamine (Flv), ketamine (Ket), vortioxetine (Vor), and trazodone (Tra), for the LPAR2-specific G protein activation and (**B**) β-arrestin recruitment. (**C**) E_{max} values (top) for each antidepressant calculated from dose-response curves presented in panels (**A**) and (**B**), and LogRAi values (bottom) for each antidepressant that showed agonist activity (E_{max} > 25% and EC_{50} < 100 μM). Data are presented as the means ± SEM. N=3. **P* < 0.05 (Unpaired t-test).

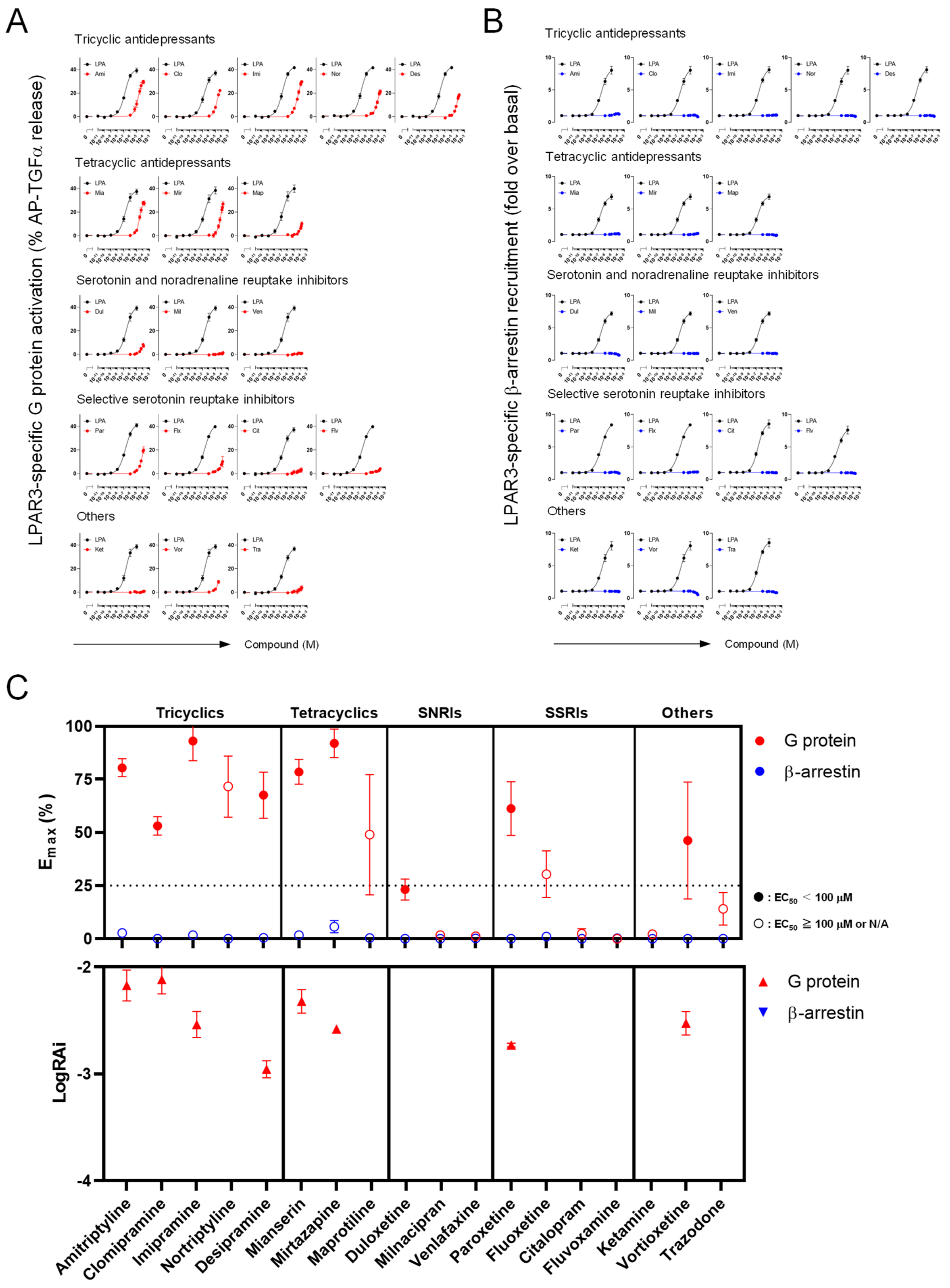

Fig. S10. Effects of various types of antidepressants on LPAR3-specific G protein and β-arrestin signaling.

(**A**) Dose-response curves of LPA and antidepressants, including amitriptyline (Ami), clomipramine (Clo), imipramine (Imi), nortriptyline (Nor), desipramine (Des), mianserin (Mia), mirtazapine (Mir), maprotiline (Map), duloxetine (Dul), milnacipran (Mil), venlafaxine (Ven), paroxetine (Par), fluoxetine (Flx), citalopram (Cit), fluvoxamine (Flv), ketamine (Ket), vortioxetine (Vor), and trazodone (Tra), for the LPAR3-specific G protein activation and (**B**) β-arrestin recruitment. (**C**) E_{max} values (top) for each antidepressant calculated from dose-response curves presented in panels (**A**) and (**B**), and LogRAi values (bottom) for each antidepressant that showed agonist activity (E_{max} > 25% and EC_{50} < 100 μM). Data are presented as the means ± SEM. N=3.

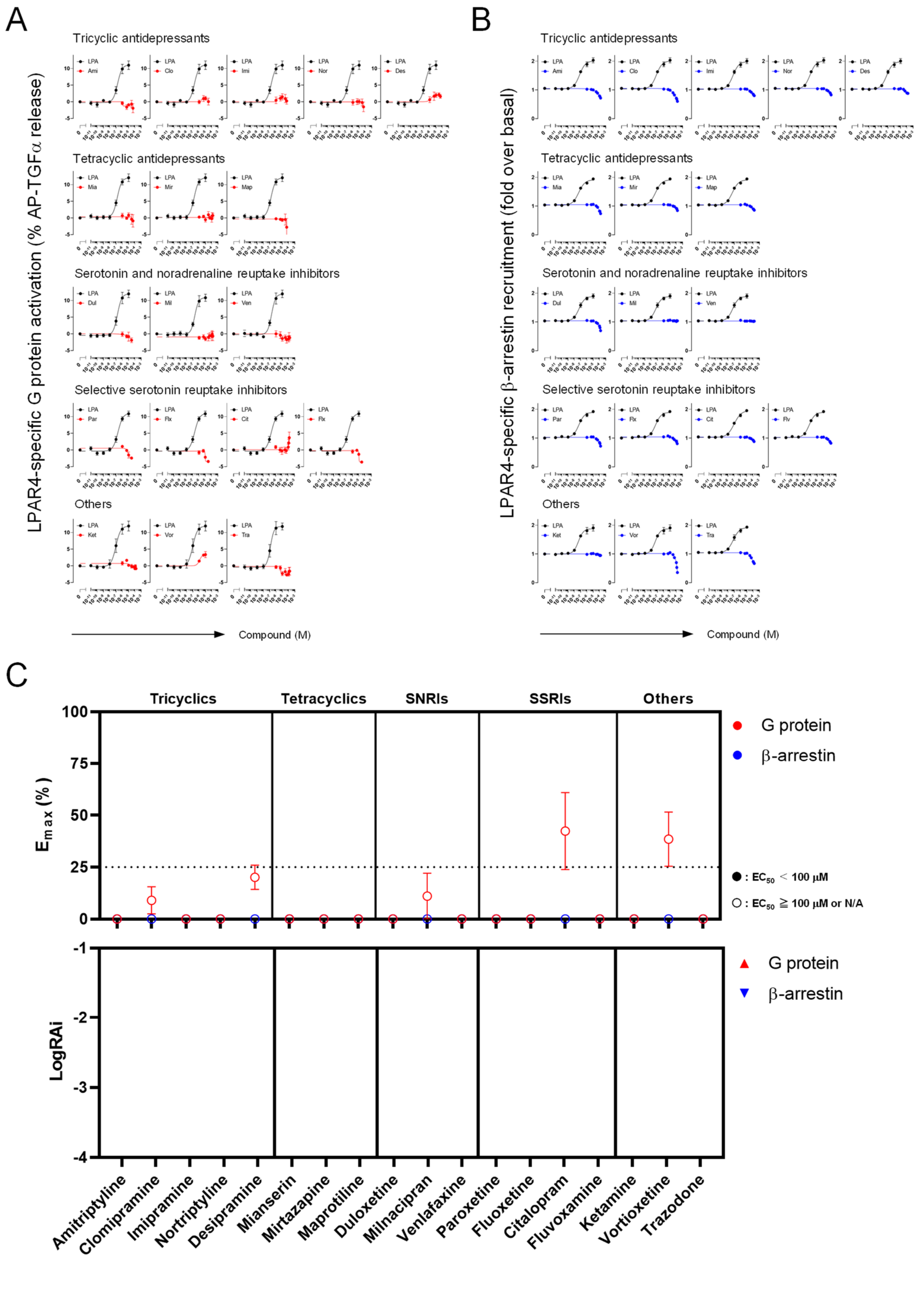

Fig. S11. Effects of various types of antidepressants on LPAR4-specific G protein and β-arrestin signaling.

(**A**) Dose-response curves of LPA and antidepressants, including amitriptyline (Ami), clomipramine (Clo), imipramine (Imi), nortriptyline (Nor), desipramine (Des), mianserin (Mia), mirtazapine (Mir), maprotiline (Map), duloxetine (Dul), milnacipran (Mil), venlafaxine (Ven), paroxetine (Par), fluoxetine (Flx), citalopram (Cit), fluvoxamine (Flv), ketamine (Ket), vortioxetine (Vor), and trazodone (Tra), for the LPAR4-specific G protein activation and (**B**) β-arrestin recruitment. (**C**) E_{max} values (top) for each antidepressant calculated from dose-response curves presented in panels (**A**) and (**B**), and LogRAi values (bottom) for each antidepressant that showed agonist activity (E_{max} > 25% and EC_{50} < 100 μM). Data are presented as the means ± SEM. N=3.

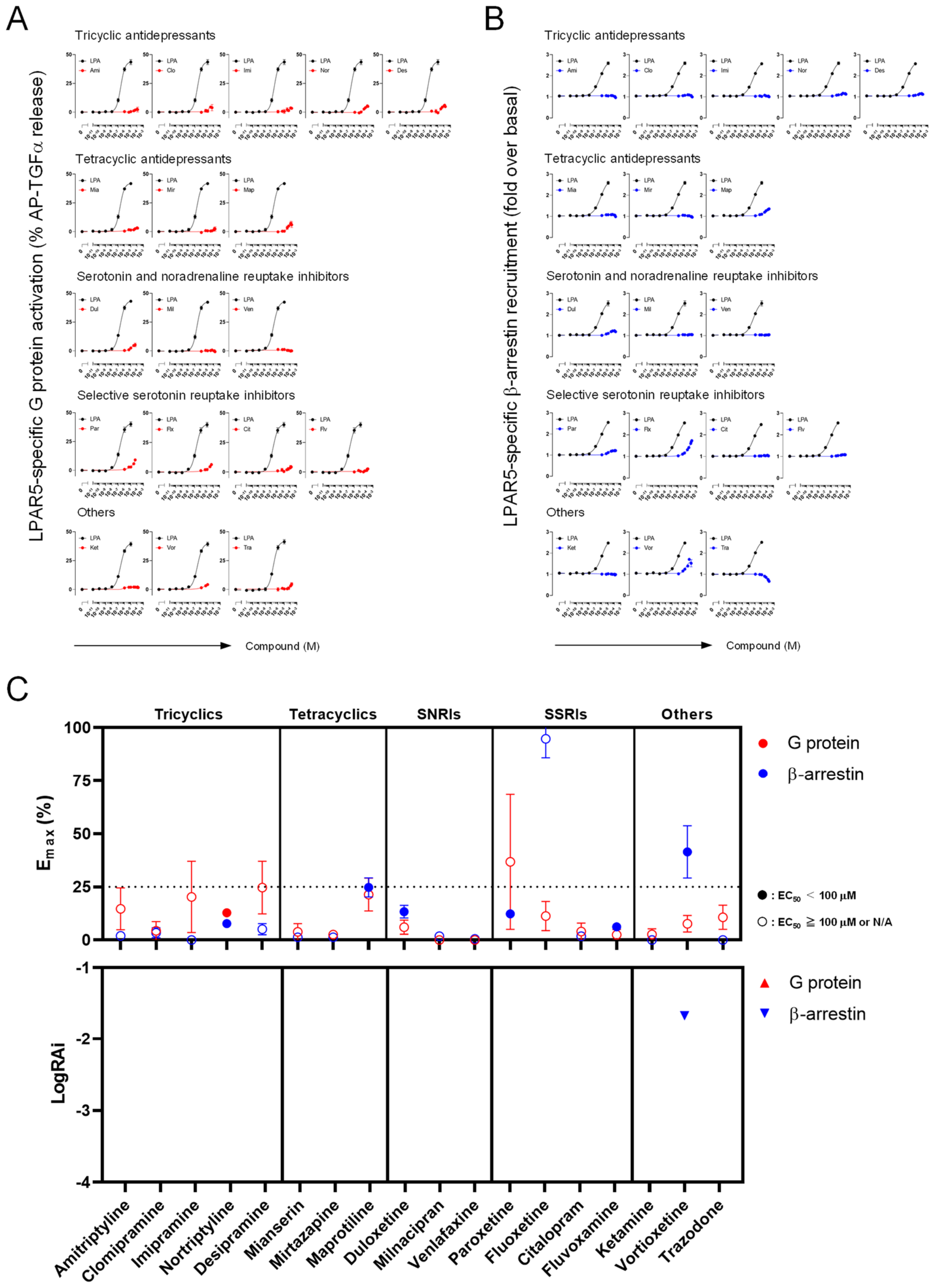

Fig. S12. Effects of various types of antidepressants on LPAR5-specific G protein and β-arrestin signaling.

(**A**) Dose-response curves of LPA and antidepressants, including amitriptyline (Ami), clomipramine (Clo), imipramine (Imi), nortriptyline (Nor), desipramine (Des), mianserin (Mia), mirtazapine (Mir), maprotiline (Map), duloxetine (Dul), milnacipran (Mil), venlafaxine (Ven), paroxetine (Par), fluoxetine (Flx), citalopram (Cit), fluvoxamine (Flv), ketamine (Ket), vortioxetine (Vor), and trazodone (Tra), for the LPAR5-specific G protein activation and (**B**) β-arrestin recruitment. (**C**) E_{max} values (top) for each antidepressant calculated from dose-response curves presented in panels (**A**) and (**B**), and LogRAi values (bottom) for each antidepressant that showed agonist activity (E_{max} > 25% and EC_{50} < 100 μM). Data are presented as the means ± SEM. N=3.

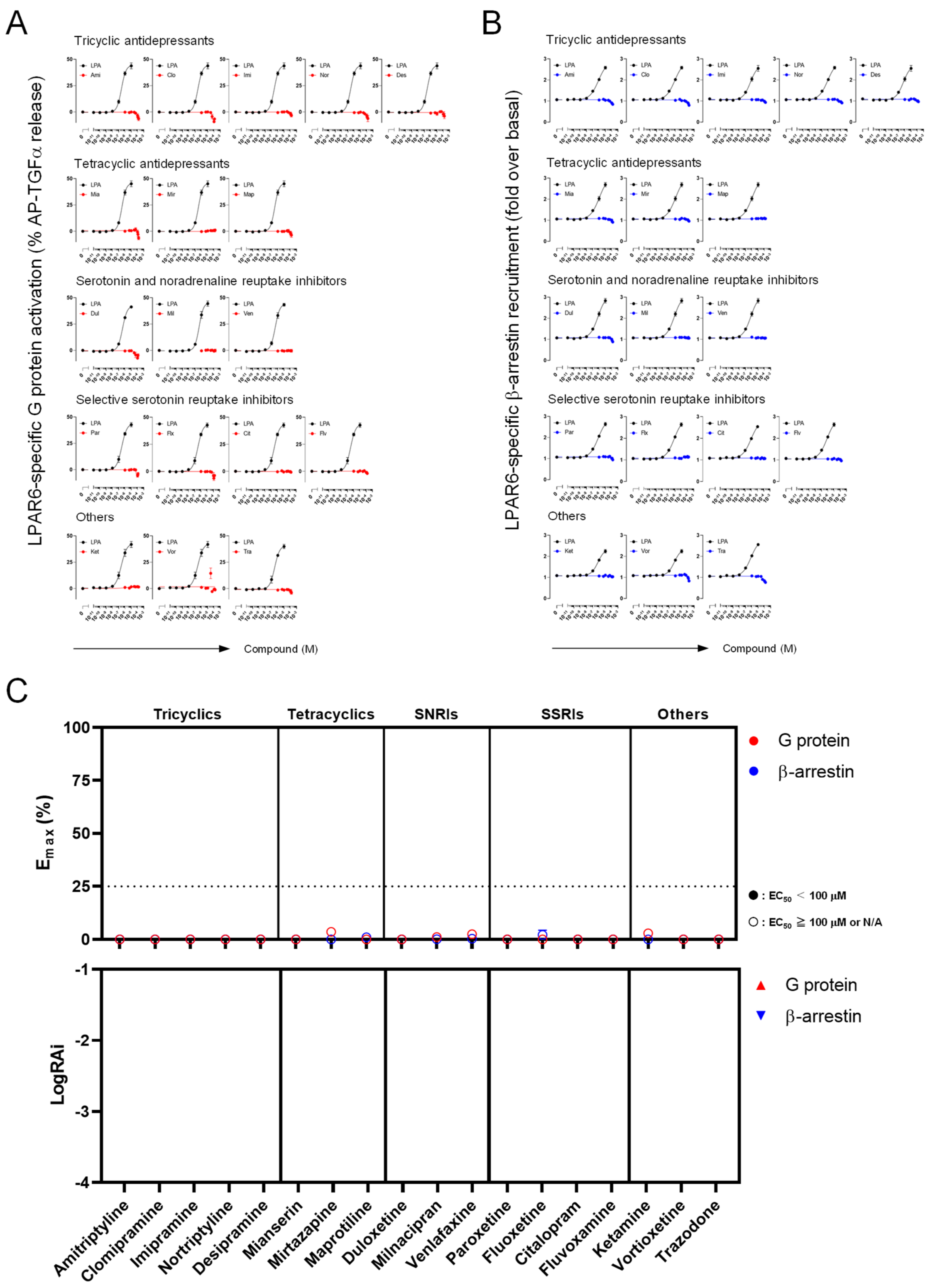

Fig. S13. Effects of various types of antidepressants on LPAR6-specific G protein and β-arrestin signaling.

(**A**) Dose-response curves of LPA and antidepressants, including amitriptyline (Ami), clomipramine (Clo), imipramine (Imi), nortriptyline (Nor), desipramine (Des), mianserin (Mia), mirtazapine (Mir), maprotiline (Map), duloxetine (Dul), milnacipran (Mil), venlafaxine (Ven), paroxetine (Par), fluoxetine (Flx), citalopram (Cit), fluvoxamine (Flv), ketamine (Ket), vortioxetine (Vor), and trazodone (Tra), for the LPAR6-specific G protein activation and (**B**) β-arrestin recruitment. (**C**) E_{max} values (top) for each antidepressant calculated from dose-response curves presented in panels (**A**) and (**B**), and LogRAi values (bottom) for each antidepressant that showed agonist activity (E_{max} > 25% and EC_{50} < 100 μM). Data are presented as the means ± SEM. N=3.

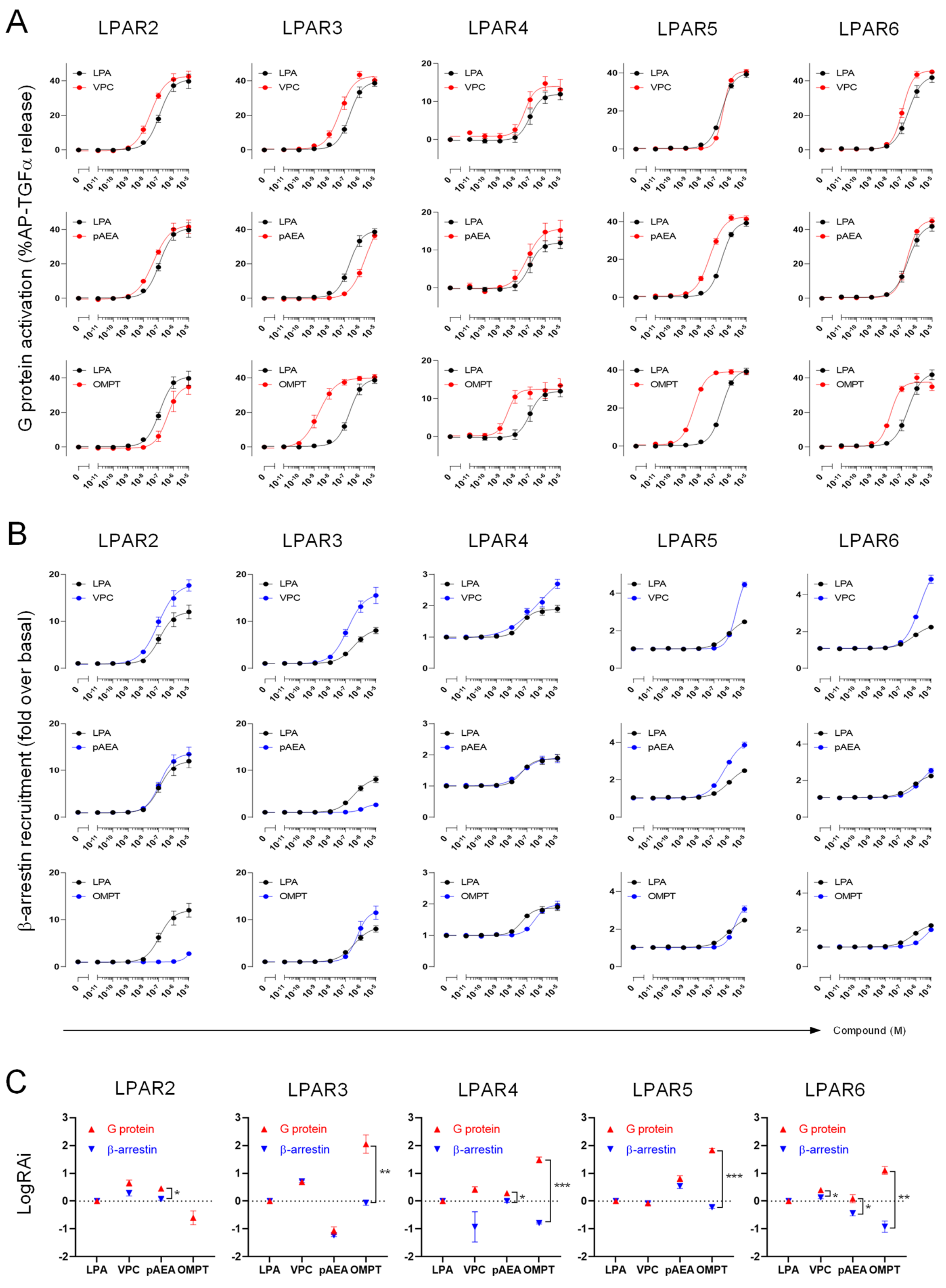

Fig. S14. Effects of LPAR agonists on LPAR2–6-specific G protein and β-arrestin signaling.

(**A**) Dose-response curves of LPA, VPC, pAEA, and OMPT for the LPAR2–6-specific G protein activation and (**B**) β-arrestin recruitment. (**C**) LogRAi values for each agonist were calculated from dose-response curves presented in panel (**A**) and (**B**). OMPT shows no agonist activity for β-arrestin recruitment to LPAR2. Data are presented as the means ± SEM. N=3. *$P < 0.05$, **$P < 0.01$, ***$P < 0.001$ (Unpaired t-test).

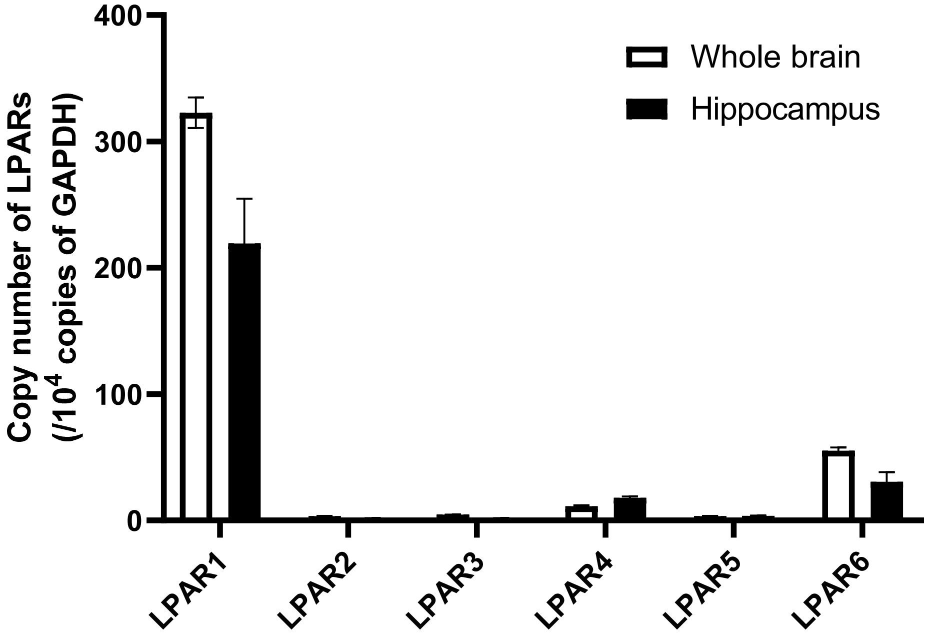

Fig. S15. Copy number of LPARs corrected against GAPDH expression in mouse whole brain and hippocampus.

Data are presented as the means ± SEM. N=3–4.

Ephrin Receptor Signaling

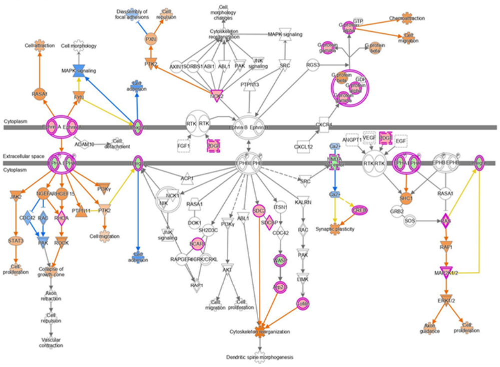

RAC Signaling

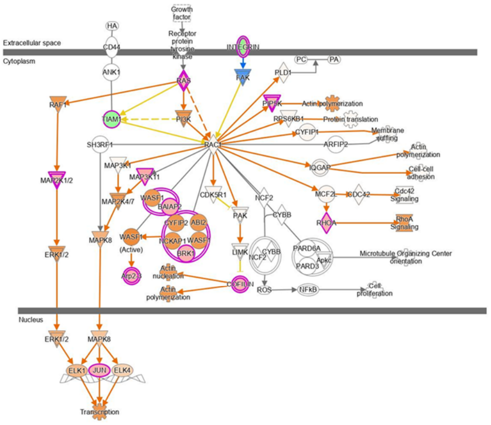

Regulation of Actin-based Motility by Rho

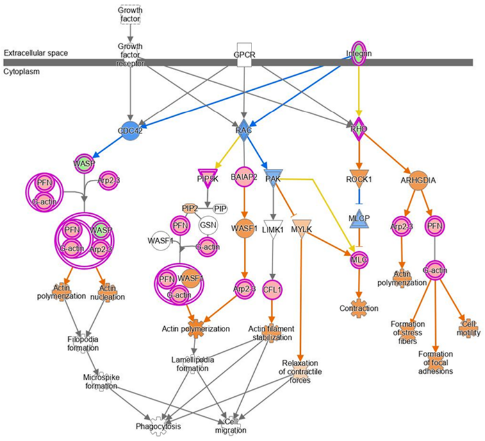

RHOA Signaling

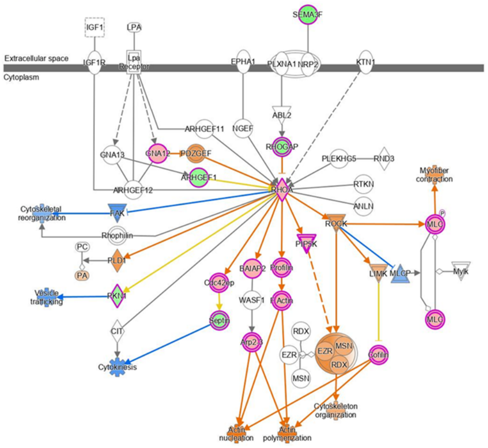

Fig. S16. Details of canonical pathways predicted to be activated by OMPT identified by ingenuity pathway analysis.

Red indicates increased measurement, orange indicates predicted activation, green indicates decreased measurement, and blue indicates predicted inhibition. Either Rho or RhoA is included in all pathways. MAP2K1/2 is included in two pathways (Ephrin Receptor Signaling and RAC Signaling).
